# Diversity and Composition of the Airborne Fungal Community in Mexico City with a Metagenomic Approach

**DOI:** 10.3390/microorganisms12122632

**Published:** 2024-12-19

**Authors:** Carolina Brunner-Mendoza, María del Carmen Calderón-Ezquerro, César Guerrero-Guerra, Alejandro Sanchez-Flores, Ilse Salinas-Peralta, Conchita Toriello

**Affiliations:** 1 Departamento de Microbiología y Parasitología, Facultad de Medicina, Universidad Nacional Autónoma de México (UNAM), Circuito Exterior s/n, Coyoacán, Ciudad Universitaria, Mexico City 04510, Mexico; brunner.carolina@gmail.com (C.B.-M.); toriello@unam.mx (C.T.); 2 Departamento de Ciencias Ambientales, Instituto de Ciencias de la Atmósfera y Cambio Climático, UNAM, Circuito Exterior s/n, Coyoacán, Ciudad Universitaria, Mexico City 04510, Mexico; cgg@atmosfera.unam.mx; 3 Unidad Universitaria de Secuenciación Masiva y Bioinformática, Instituto de Biotecnología, UNAM, Cuernavaca 62210, Morelos, Mexico; alejandro.sanchez@ibt.unam.mx (A.S.-F.); ilse.salinas@ibt.unam.mx (I.S.-P.)

**Keywords:** metagenomics, airborne, fungi, diversity, seasonality

## Abstract

Airborne fungi are widely distributed in the environment and originate from various sources like soil, plants, decaying organic matter, and even indoor environments. Exposure to airborne fungal spores can cause allergic reactions, asthma, and respiratory infections. Certain fungi can cause serious infections, particularly in individuals with weakened immune systems. An aerobiological study was conducted to detect airborne fungi using metagenomics in three areas of Mexico City, each representing different degrees of urbanization, during the dry and rainy seasons of 2017. Seasonality showed a significant role in the composition of airborne fungi. Ascomycota predominated in the three areas sampled during dry and rainy seasons, with the genera *Cladosporium* and *Penicillium* presenting the highest relative abundances across seasons. The Agaromycetes showed an increase during the rainy season. Regarding the areas, the north and center sites showed similar meteorological conditions and fungal community composition patterns. According to the Shannon and Simpson indices, the south area showed a greater species diversity during the dry season. These insights into the interactions between urbanization, seasonality, and airborne microbial communities could contribute to more effective urban management, reduced health risks, and the promotion of sustainable development.

## 1. Introduction

Fungal presence in the atmosphere is common due to several factors related to fungi’s life cycle and ecological strategies [1]. Surrounding sources continually release propagules into the atmosphere, shaping the diversity and abundance of airborne fungal communities [2]. The dispersion of fungi is a complex process driven by a combination of biological, environmental, and anthropogenic factors [3]. Since the emergence and global spread of COVID-19, bioaerosols have received considerable attention. Fungi constitute a significant component of bioaerosols, alongside pollen, bacteria, algae, viruses, and fragments from animals and plants [4,5,6]. Currently, diverse collection methods (such as sedimentation, filtration, centrifugation, impaction, impingement, and microfluidic methods) and detection methods (such as cultures, molecular biological assays, and immunological assays) have been developed to characterize bioaerosols [7]. Metagenomic tools have become a driving force for discoveries in microbial ecology and biotechnology, serving as a critical method for exploring the microbial universe [8]. Through metagenomic analysis, fungal communities have been characterized in various settings including urban and rural areas as well as indoor and outdoor environments [9,10]. Additionally, seasonality patterns and the relationship between mycobiomes and particulate matter have been analyzed [11]. Unlike other major public health pollutants, bioaerosol surveillance has been underestimated, despite studies highlighting their relevance and their role as enhancers of allergic diseases through co-exposure to these pollutants [12]. Furthermore, airborne fungal pathogens are associated with life-threatening primary and secondary infections in immunocompromised patients [13,14].

Moreover, the unregulated development of urban spaces, with little regard for environmental protection, has caused demographic centralization, severe traffic congestion, energy resource shortages, ecological deterioration, and public health impacts [15]. Previous studies in Mexico City have monitored the microbial community composition and its associations with meteorological factors, land use changes, particulate matter, and pollutants like ozone. These studies reveal shifts in the microbial community composition, primarily influenced by seasonality, with subtle changes linked to the location. They also highlight the potential health risks posed by some airborne microorganisms [16,17,18].

Mexico City, one of the largest and most polluted megacities in the world, exhibits a complex interplay of environmental and anthropogenic factors that could shape the diversity and distribution of airborne fungi. Air pollution, temperature fluctuations, and humidity levels interact to create an environment that supports the proliferation of some fungal species while suppressing others. These factors, coupled with seasonal variations, make Mexico City a compelling study area for investigating airborne fungal communities [16]. Understanding the composition and dynamics of these communities is essential for assessing their potential impact on public health, biodiversity, and air quality.

To enhance bioaerosol surveillance in Mexico City and investigate the fungal community composition in response to seasonality, green areas, and meteorological factors, we conducted an aerobiological study. This study focused on the metagenomic detection of airborne fungi in three distinct areas of Mexico City, each representing different levels of urbanization, during the dry and rainy seasons of 2017. This information can be used to evaluate the state of urban ecosystems, predict responses to environmental changes, develop strategies for the conservation and sustainable management of natural resources, and identify pathogens impacting human and environmental health.

## 2. Materials and Methods

### 2.1. Sampling Area

**Figure 1:**
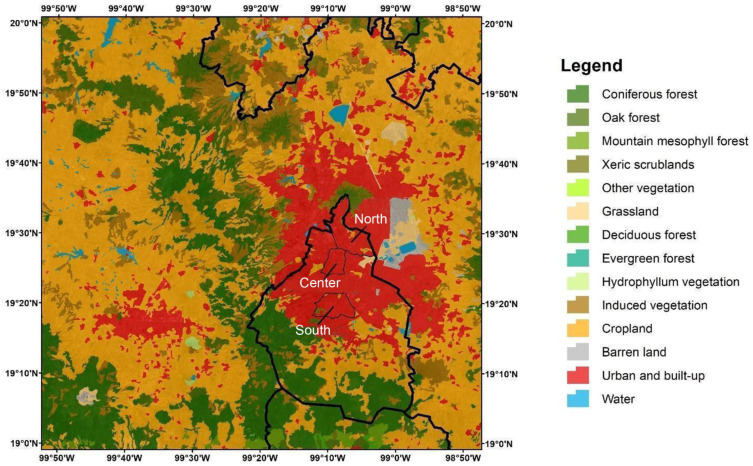
Location of aerobiological stations in the south, center, and north of Mexico City and the different land uses in the city.

The study was conducted in Mexico City in three locations (2240 m.a.s.l.) during the dry season (January 3 to April 30) and the rainy season (August 1 to October 29) of 2017. An aerobiological monitoring station was located in the north part of Mexico City at 19°30′43”N, 99°08′16”W (2240 m.a.s.l.), on the rooftop (15 m high) of the ‘Escuela de Medicina y Homeopatía’ building on the Instituto Politécnico Nacional (IPN) campus. Another station was located in the center area of Mexico City, at 19°26′08”N, 99°08′22”W, on the rooftop (15 m high) of the ‘Palacio de Minería’ building. The final station was located in the south part of the city at 19°32′62”N, 99°17′61”W, on the rooftop (15 m high) of the ‘Instituto de Ciencias de la Atmósfera y Cambio Climático,’ on the Universidad Nacional Autónoma de México (UNAM) campus (Figure 1 and Table 1).

Meteorological parameters were selected to characterize each zone and identify differences between the dry and the rainy seasons. The daily average temperature, relative humidity, and accumulated precipitation were collected. These data were obtained from the Red Universitaria de Observatorios Atmosféricos (RUOA network: www.ruoa.unam.mx (accessed on 5 September 2024)).

### 2.2. Air Bioaerosol Sampling

The samples were collected using high-volume samplers (PM_10_) (GMW Model 1200, VFC HVPM10; Sierra Andersen, Smyrna, GA, USA) with an airflow rate of 1.13 m^3^/min. Nitrocellulose membranes (11302-131, Sartorius, Göttingen, Germany) previously sterilized with ultraviolet light for 30 min were used to capture them. The samplers were cleaned with alcohol (70%) before and after each sampling. Samples were collected three times a week for 24 h periods. Filters were collected from the sampler every week after 72 h of sampling. The filters were handled using sterile nitrile gloves and N95 masks, wrapped in sterile aluminum foil, placed in sterile envelopes, and stored at −70 °C until use. Particles were removed from the membranes by carefully sweeping them with a fine brush or scalpel, both of which were previously sterilized with ultraviolet light for 30 min. The particles were then collected in sterile glass vials, weighed, covered with aluminum foil, and stored in a plastic container at −70 °C until use. All glass bottles used for particle collection and storage were washed with 10% Extran^®^ for 24 h, rinsed with running water followed by distilled water, sterilized in an autoclave, and dried at 250 °C for 1 h. 

### 2.3. DNA Extraction

The samples were processed in a laminar flow cabinet, which was previously cleaned with a 0.1% benzalkonium chloride solution and sterilized using ultraviolet (UV) radiation. For the extraction of metagenomic DNA, 10 mg of particles were resuspended in 2 mL tubes with screw caps. Each tube contained 400 µL of an extraction buffer (0.1 M Tris-HCl, pH 7.5; 0.05 M EDTA (Ethylenediaminetetraacetic acid), pH 8.0; 1 M KCl; and 0.1% Nonidet P40). Genomic DNA was extracted using the Fast DNA Spin Kit for Soil (MP BIOMEDICALS, Irvine, CA, USA) following the manufacturer’s instructions. Three replicates of each sample (250 μL) were processed in 2 mL tubes with a screw cap. Two elutions were performed using nuclease-free water, each with a volume of 50 μL, to maximize the DNA yield. The elutions were concentrated to approximately 25 μL using a SpeedVac Concentrator (DNA120 Savant equipment, Thermo Scientific, Waltham, MA, USA).

The genomic DNA quantification and purity were determined using, 2 µL of the sample with a Qubit fluorometer (Thermo Fisher Scientific, USA). The DNA was sent to Macrogen (Macrogen Inc., Seoul, Republic of Korea) for ITS rRNA library construction. The ITS region was amplified using the primers ITS3_KY02 (GATGAAGAACGYAGYRAA) and ITS4 (TCCTCCGCTTATTGATATGC) [19] according to Illumina protocol. The prepared libraries were sequenced in an Illumina MiSeq system (Illumina Inc., San Diego, CA, USA). Macrogen Inc. performed taxonomic analysis using their validated protocols, as outlined in the NGS Analysis Manual for OUT classification. The analysis utilized QIIME to assign a taxonomy based on representative sequences from each OTU, accompanied by statistical and phylogenetic evaluations. 

All sequencing data supporting this study’s findings are available at the National Center for Biotechnology Information (NCBI) under submission ID SUB14752941 and BioProject ID PRJNA1165756.

### 2.4. Bioinformatic Analysis

The original amplicon region was reconstructed by overlapping paired-end reads using Flash version 1.2.11 [20]. Merged paired sequences were used as the input for taxonomic annotation performed with Parallel-Meta version 2.4.1 [21] against the Metaxa2 database version 2.1.1 [22], as described by [23]. Statistical analyses and plotting were performed using R Statistical Software (v4.3.2; R Core Team 2023) and associated packages [24].

Data manipulation, barplot creation, and diversity index calculations were performed using the phyloseq package [25] (https://github.com/joey711/phyloseq, accessed on 24 August 2023). Rarefaction curves were generated with the ranacapa package [26] (https://github.com/gauravsk/ranacapa, accessed on 24 August 2023), and statistical differences between Shannon indexes were calculated using the ggpubr package [27]. (https://cran.r-project.org/web/packages/ggpubr/index.html, accessed on 7 November 2023; PCA analysis was performed and visualized with the ade4 package [28]. (https://adeverse.github.io/ade4/, accessed on 6 January 2024), while heatmaps and Venn diagrams were created using ggplot2 (https://cran.r-project.org/web/packages/ggplot2/index.html, accessed 6 January 2024), and (https://github.com/NicolasH2/ggvenn, accessed 6 January 2024), with the given package [29,30].

## 3. Results

### 3.1. Meteorological Parameters

The center site exhibited the highest temperatures during the dry season. The south site showed the most significant variation in the relative humidity between seasons. The north site recorded the lowest precipitation levels (Table 2).

### 3.2. Sequencing Data

A total of 72 air samples were obtained. After removing ambiguous, low-quality, denoising, and chimera sequences, the total number of sequence reads was 2,354,249, with a gamma diversity of 5753. At the phylum level, the taxonomic assignments were Eukaryota (5.3%), Ascomycota (72.4%), Basidiomycota (8.1%), Chlorophyta (0.2%), Streptophyta (12%), and unassigned (2.1%). 

The highest TTa values (1,233,856) were recorded at the south site during the dry season. At the genus level, slightly higher TTaG values were observed at the center site during the dry season (Table 3).

**Figure 2:**
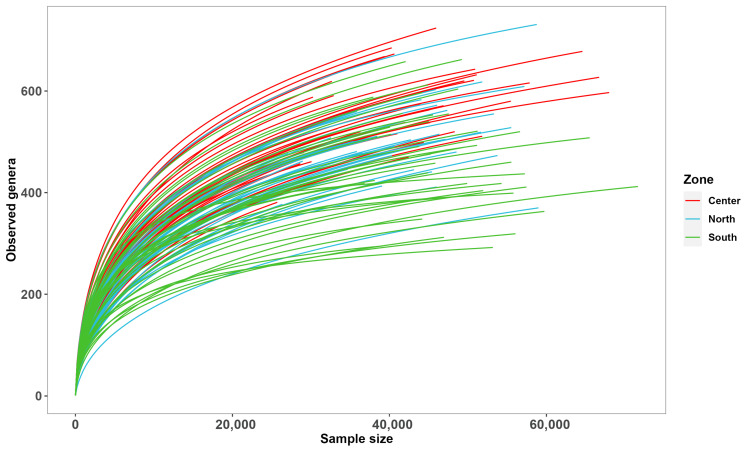
Rarefaction curve of the observed taxonomic units (TTas) of fungal taxa in the three sample zones.

According to the rarefaction curve (Figure 2), the fungal diversity across seasons and zones was adequately characterized during the study. The bioinformatics analysis of the (fungal) ITS indicated, through the rarefaction curves, that the bioaerosol sampling and DNA detection achieved the required sampling depth for molecular rarefaction curves to estimate the expected number of species for a given sample size based on a hypergeometric distribution. As shown in Figure 2, most samples approach a plateau, indicating that the sequencing depth achieved is sufficient for our analysis.

### 3.3. Fungal Community Composition

**Figure 3:**
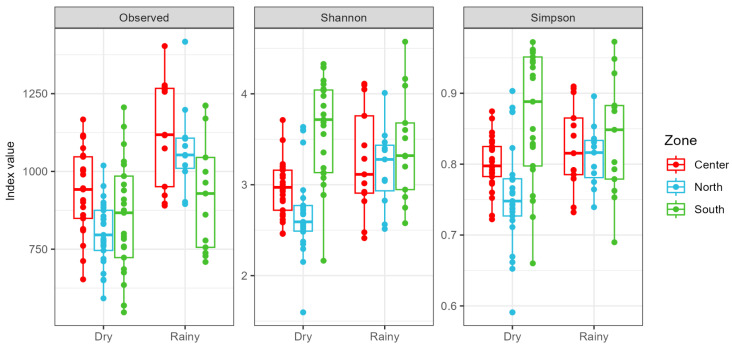
Alpha diversity indices (TTas, Shannon, and Simpson) across different zones and seasons. Boxes represent the interquartile range, horizontal lines within the boxes represent the median, and dots represent individual data points.

According to the Shannon and Simpson indices, the south site exhibited greater species diversity in both seasons (Figure 3).

During the dry season, the center and south sites exhibited higher diversity compared to the north site, while in the rainy season, the south site had the highest diversity. 

**Figure 4:**
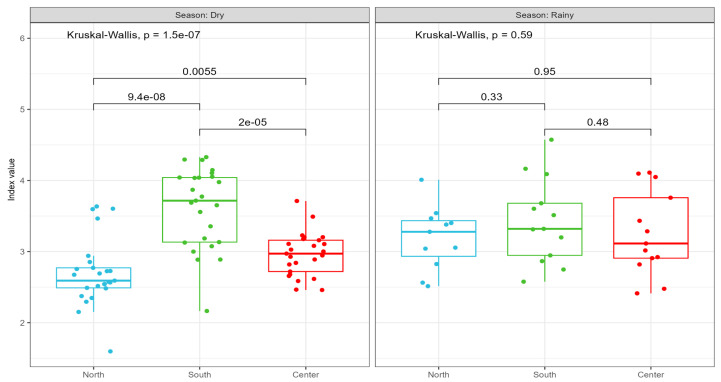
Comparison of Shannon index across different zones and seasons. Box plots show the median (horizontal line within the box), interquartile range (box), and individual data points (dots), indicating the variability within each zone for both seasons. Blue = north; green = south; and red = center.

In the dry season (Figure 4, left panel), the Kruskal–Wallis test showed a significant difference between sites (*p* = 1.5e-07). Pairwise comparisons showed significant differences between the north site and south site (*p* = 9.4e-08), the north and center sites (*p* = 0.0055), and the south and center sites (*p* = 2e-05). In the rainy season (Figure 4, right panel), the Kruskal–Wallis test showed no significant differences between sites (*p* = 0.59). Pairwise comparisons also showed no significant differences between the north and south sites (*p* = 0.33), the north and center sites (*p* = 0.95), and the south and center sites (*p* = 0.48).

In summary, during the dry season, Shannon index values varied significantly across the three zones, with the north site showing the lowest diversity and the south site the highest. During the rainy season, no significant differences were observed between the groups, and the diversity values were similar across the three regions.

### 3.4. Main Groups of Airborne Fungi Identified in the South, Center, and North of Mexico City

The phylum Ascomycota predominated throughout the sampling period, with higher percentages (80% to 90%) during the dry season across the three zones. The phylum Basidiomycota was the second most abundant, increasing during the rainy season (17% to 26%) compared to the dry season (10% to 18%) (see Table 4 and Supplementary Material Figures S1 and S2).

Within the Ascomycetes, Dothideomycetes were highly represented, reaching the highest percentages at the center site during both the dry (79%) and rainy (73%) seasons (Figure 1 and Figure 2).

**Figure 5:**
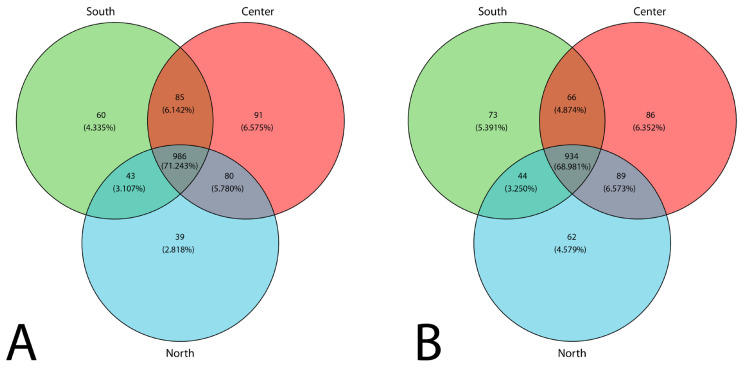
Venn diagram of airborne fungal diversity (TTaGs) based on the ITS rRNA region across the three sampled zones: (**A**) dry season, (**B**) rainy season.

A Venn diagram (Figure 5A, B) illustrates the distribution of fungal taxa across the south, center, and north sites. It depicts the relative abundance of the TTaGs in each sampled area. In both seasons, the center site recorded a higher number of unique genera, which were not observed at the south or north sites.

A set of fungal taxa was consistently present throughout the sampling period. *Cladosporium* exhibited the highest relative abundances at all three stations during the dry season, reaching 40% and 45% at the north site during the rainy and dry seasons, respectively. This was followed by *Ascochyta*, which recorded relative abundances of 16% and 18% at the center site during the rainy and dry periods, respectively. *Alternaria* was present in an 8% and 9% relative abundance at the center and north sites across both seasons. In contrast, *Aspergillus* and *Penicillium* varied across all monitored areas, with relative abundances ranging from 1% to 5% in both seasons. Among the Basidiomycota, *Quambalaria* was prominent during the dry season in all three zones, with relative abundances of 5% at the center site, 8% at the north site, and 3% at the south site (Supplementary Material Figures S1 and S2).

**Figure 6:**
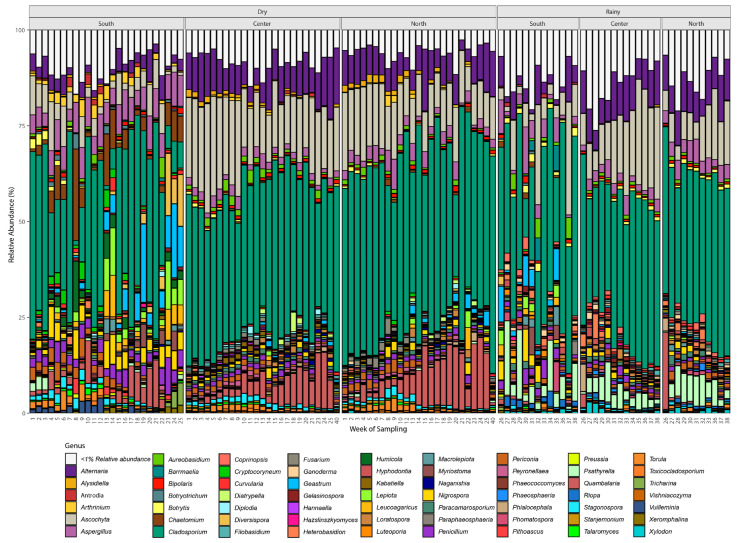
Airborne fungi community diversity and composition in the north, central, and south zones.

Figure 6 illustrates the relative abundance of fungal genera detected in three distinct locations, the south, center, and north of the study area, across two seasons, the dry season and the rainy season. The x-axis represents the week of sampling, while the y-axis shows the relative abundance (%) of fungal genera. Each bar represents a specific sampling week, with colors indicating different fungal genera, as shown in the legend. In both the dry and rainy seasons, the fungal community composition exhibited notable changes. Some genera dominated during specific weeks and seasons, reflecting seasonal shifts in fungal diversity and abundance. Location-specific patterns are evident; for example, the south exhibited higher fungal diversity during both seasons compared to the north and center. Genera such as *Cladosporium, Alternaria, Aspergillus,* and *Penicillium* were among the most abundant across all sites, with fluctuations in relative abundance over weeks and seasons. Some less abundant genera contributed to the overall diversity but did not dominate in terms of relative abundance. The Agaricomycetes increased during the rainy season, with *Psathyrella purpureobadia* showing the highest relative abundance (4%, 5%, and 6% at the center, north, and south sites, respectively). Other genera, including *Lepiota*, *Leucoagaricus*, *Antrodia*, *Ganoderma*, *Geastrum*, *Heterobasidion*, *Filobasidium*, and *Naganishia*, were present with relative abundances below 1% (Figure 6, Supplementary Material Figure S2).

### 3.5. Taxonomic Approximation at the Species Level

At the species level, the global analysis identified several pathogenic fungi, including *Cladosporium cladosporioides, Alternaria alternata, Aspergillus fumigatus, Aureobasidium pullulans, Mucor circinelloides,* and *Curvularia lunata*. An important finding at the south station was the detection of *Coccidioides posadasii*. This was observed during 21–27 February 2017 (mean temperature of 16 °C; RH% of 38.1; accumulated rain of 0 mm) and 21–27 March 2017 (mean temperature of 17.2 °C; RH% of 41.7; solar radiation of 11,746.1; accumulated rain of 2.4 mm). Additionally, several phytopathogenic species of agricultural concern were detected, including *Golovinomyces cichoracearum, Alternaria alternata, Colletotrichum gloeosporioides,* and *Fusarium oxysporum*.

## 4. Discussion

Fungi present in the atmosphere are dispersed, using the atmosphere as transport rather than a habitat [31]. Several studies have analyzed the diversity of airborne fungi in outdoor environments, providing insights into biological air quality and airborne disease prevention [32,33,34].

Consistent with previous studies, the phylum Ascomycota predominated across different sampling methods, seasons, and land uses (urban, rural, agricultural, forestry, marine) [16,17,35,36,37,38]. Universally distributed fungal groups, *Cladosporium*, *Aspergillus*, *Penicillium*, and *Epicoccum*, were observed [17,18,37,39,40].

The widespread presence of *Cladosporium* has been attributed to its small conidia that easily spread over long distances [41,42] and its high adaptability to survive as a saprophyte, pathogen, and endophyte and in extreme habitats. Additionally, *Cladosporium* comprises complex and diverse species, with approximately 772 taxonomic records, a number likely to grow due to ongoing isolations from various sources, including plants, water, air, food, soil, and clinical samples. Although *Cladosporium* is widespread and generally poses a low health risk, continuous monitoring is advisable. Studies, such as that by Ballero et al. [43], indicate that even a concentration of 100 spores/m^3^ can initiate early allergic reactions.

Our results show that the composition of airborne fungi communities varies significantly, with seasonality being a key factor. Seasonality plays a significant role in shaping the composition of airborne communities by influencing environmental conditions, vegetation dynamics, weather patterns, and human activities [38,42]. An increase in basidiomycetes during the rainy season and a greater fungal richness during the dry season were recorded. Previous studies have reported higher fungal concentrations in summer or late summer and early autumn, particularly when rainy days are followed by sunny, dry, and windy conditions [38]. 

The differences in relative abundances observed at the three sampling sites could be attributed to the dispersion of fungal propagules, influenced by the predominant wind direction in Mexico City [44]. Average wind speeds in the city are generally moderate, ranging from 2 to 4 m/s (meters per second), with higher speeds occurring during cold fronts or storms [45]. These wind patterns can transport fungal spores from surrounding forests, grasslands, and agricultural areas into the urban environment. The basin-like topography of Mexico City contributes to the accumulation of fungal spores in certain areas, particularly during calm wind conditions, creating localized hotspots of airborne fungi. During the dry season, stronger winds facilitate the transport of spores from natural and agricultural areas into the city, potentially enhancing the diversity of airborne fungal communities [44].

Fungal communities in Mexico City are influenced by both natural sources, such as vegetation (e.g., forests), and anthropogenic sources, including urban environments like construction sites and waste. The wind-driven dispersal of allergenic fungi, including *Cladosporium*, *Alternaria*, *Aspergillus*, and *Penicillium*, can peak during specific seasons, potentially exacerbating seasonal allergies and respiratory issues. 

BLAST analysis revealed the presence of highly relevant pathogenic species, such as *Coccidiodes posadasii.* However, this pathogen was detected in very low relative abundances, appearing only at the south site during certain days in February and March 2017. These findings highlight the need for further research and the targeted monitoring of this pathogen to understand its sources, evaluate its concentrations, and determine its viability. Recovering this fungus from soil through cultivation is challenging, as it occurs in low proportions in soil samples from endemic regions [46]. 

*Cladosporium*, *Alternaria*, *Aspergillus*, and *Penicillium* were constantly found in our study. These genera are among the most common fungal allergens, along with over 80 fungal genera known to induce type I allergies in susceptible individuals [47].

The presence of airborne fungal genera in outdoor environments can significantly impact human health, particularly for individuals with respiratory conditions, allergies, or compromised immune systems [48]. *Cladosporium*, one of the most abundant fungal genera in the atmosphere, is a common allergen that can trigger asthma flares and allergic rhinitis upon the inhalation of its spores [41]. *Alternaria* is similarly associated with respiratory allergies and is a major contributor to allergic asthma, often exacerbating symptoms during its peak seasons [42]. *Aspergillus*, which includes species like *A. fumigatus*, is a notable opportunistic pathogen; its spores can cause allergic bronchopulmonary aspergillosis (ABPA) or invasive infections in immunosuppressed individuals [49]. Meanwhile, *Penicillium*, although primarily associated with indoor environments, is also found outdoors and can produce spores that exacerbate asthma and allergies [50]. Additionally, certain species of *Aspergillus* and *Penicillium* are capable of producing mycotoxins, which can have toxic effects on humans upon prolonged exposure [48]. Furthermore, bioaerosols can interact with chemical pollutants, producing synergistic effects that worsen health impacts, especially on respiratory and cardiovascular systems [51].

Hypersensitivity reactions associated with fungal allergens include rhinitis, asthma, atopic dermatitis, and allergic bronchopulmonary mycoses [52]. In addition to causing hypersensitivity reactions, many airborne fungi are capable of causing severe diseases. Estimates of the incidence and prevalence of severe fungal infections (SFIs) in Mexico highlight the significant burden of airborne fungi-related diseases, including allergic bronchopulmonary aspergillosis (60 per 100,000), chronic pulmonary aspergillosis (15.9 per 100,000), coccidioidomycosis (7.6 per 100,000), and invasive aspergillosis (4.56 per 100,000) [53].

Besides the importance of air biomonitoring to inform the public about the presence of allergy- and disease-causing fungi, understanding the composition and diversity of airborne fungi is equally crucial. Many fungal species directly affect ecosystem services and human well-being [54]. Some studies have revealed the relationship between urban green spaces and healthier environments and lifestyles [55].

In recent years, understanding the impact of urbanization has become increasingly important due to its relationship with climate change and biodiversity, which are key priorities for sustainable development [42]. In this context, the north and center sites exhibit similar patterns in terms of the temperature, precipitation, and humidity. However, they differ significantly in the green area coverage: the north site has the highest percentage of green areas, while the center site has the fewest. According to alpha diversity indices, the south site shows the highest values. Although this area does not surpass the north site in terms of total green areas, its green areas are more evenly distributed. In contrast, the north site’s green areas are concentrated in a single location, the Sierra de Guadalupe State Park.

Interestingly, the center site, despite having the lowest percentage of green areas, recorded the highest total taxonomy annotations (TTas) during the dry season. This is explained by the fact that TTas reflect the total number of species without considering species evenness. In comparison, the Shannon and Simpson indices, which consider both evenness and relative abundance, typically report lower values for the center site. Moreover, biodiversity is not always a straightforward indicator of environmental health; complex interactions between diversity and disturbances may reflect responses to environmental fluctuations [56].

Understanding these complex interactions between urbanization, seasonality, and airborne microbial communities is essential for managing urban environments, mitigating health risks, and promoting sustainable development practices.

## 5. Conclusions

This study highlights the importance of the fungal composition and diversity in Mexico City’s air, revealing Ascomycota to be the dominant phylum across environments and seasons. Seasonal changes influence fungal communities, with an increase in basidiomycetes during the rainy season and a higher species richness in the dry season. Genera such as *Cladosporium*, *Aspergillus*, and *Penicillium* are prevalent in urban areas and are associated with allergies and respiratory diseases. The continuous monitoring of these fungi is crucial for understanding their impact on public health, emphasizing the need for comprehensive aerobiological studies to mitigate health risks and improve urban air quality. Likewise, the south site presents a greater diversity of species than the other zones. Although the center zone has few green areas, its biodiversity is notable; however, this does not always translate into better environmental well-being. On the other hand, the north and center areas share similar meteorological and fungal composition patterns, highlighting differences in each region’s ecological dynamics. These complex interactions between urbanization, seasonality, and microbial communities are essential for health risk management and sustainable development in urban environments.

## Figures and Tables

**Table 1:** Characteristics of the sampling areas.

Zone	Urban Area * %	Green Area * %	Number of Inhabitants **	Population Percentage ***	Area (km^2^) **	Population Density ****
North site	83.53	16.31	1,173,351	12.74	87.38	72.9
South site	93.05	6.95	620,416	6.74	53.62	49.9
Center site	100	0.00	545,684	5.92	32.24	32.24

Sources: Agustín Fernández, UNIATMOS, ICAyCC. * Calculated by UNIATMOS from the set of vector data on land use and vegetation; a scale of 1:250,000. SERIES VII. National set (INEGI, 2018). ** Taken from the municipal reference layer (Informatics Unit for Atmospheric and Environmental Sciences, 2020). *** Number of inhabitants of each municipality in relation to the total number of inhabitants of Mexico City: 9,209,944 (INEGI, 2020; https://www.inegi.org.mx. (accessed on 10 December 2020) **** Area in km^2^ of each territorial demarcation by the percentage of land use/100 (INEGI 2020

**Table 2:** Climatic conditions across south, center, and north sites during dry and rainy seasons.

	South Site				Center Site				North Site			
	Dry Season	SD	Rainy Season	SD	Dry Season	SD	Rainy Season	SD	Dry Season	SD	Rainy Season	SD
Average Temperature (°C)	16.2	2.2	15.9	1.7	17.7	2.3	17.4	1.5	16.6	2.3	16.8	1.5
Maximum Temperature (°C)	23.7	2.3	22.7	2.5	24.5	2.5	23.6	2.2	24.5	2.4	23.4	2.3
Minimum Temperature (°C)	9.2	2.4	10.6	1.9	11.8	2.4	12.6	1.6	9.3	2.4	11.0	1.8
Relative Humidity (%)	47.8	11.6	63.3	10.6	45.4	11.6	61.2	9.5	46.9	11.9	62.5	10.1
Average Precipitation (mm)	0.3	1.3	1.3	6.5	0.2	0.9	1.5	5.2	0.2	0.9	1.6	6.9

**Table 3:** Characteristics of the metagenomic analysis (ITS clone libraries) across seasons and zones.

Characteristic	Center Site		North Site		South Site	
Dry	Rainy	Dry	Rainy	Dry	Rainy	
Raw reads	1,261,767	676,479	1,238,508	568,477	1,445,707	650,097
Post-filtering	1,260,162	675,577	1,236,925	567,806	1,444,137	649,329
Post-merging reads	1,224,560	660,289	1,201,360	556,023	1,393,755	634,446
Total taxonomy annotations (TTas)	998,114	633,457	953,462	543,263	1,233,856	623,805
Total taxonomy annotations at genus level (TTaGs)	1242	1175	1148	1129	1174	1117
Unclassified taxa at genus level	4	4	4	3	4	2
Classified taxa at genus level	1238	1171	1144	1126	1170	1115
Uniqueknown genera	91	86	39	62	60	73

**Table 4:** Main fungal phylum and class identified across seasons and zones.

	Dry Season			Rainy Season		
Phylum	South	Center	North	South	Center	North
Ascomycota	80	90	86	74	83	80
Basidiomycota	18	10	13	26	17	20
Mucormycota	2	---	--			

## Data Availability

The original contributions presented in this study are included in the article/Supplementary Materials; further inquiries can be directed to the corresponding authors.

## Supplementary Materials

The following supporting information can be downloaded at: https://www.mdpi.com/article/10.3390/microorganisms12122632/s1.

## References

[1] Hernandez H., Martinez L.R. (2018). Relationship of environmental disturbances and the infectious potential of fungi. Microbiol. Read..

[2] Bowers R.M., Clements N., Emerson J.B., Wiedinmyer C., Hannigan M.P., Fierer N. (2013). Seasonal variability in bacterial and fungal diversity of the near-surface atmosphere. Environ. Sci. Technol..

[3] Li D.W., Yang C.S. (2004). Fungal contamination as a major contributor to sick building syndrome. Adv. Appl. Microbiol..

[4] Despres V.R., Huffman J.A., Burrows S.M., Hoose C., Safatov A.S., Buryak G., Fröhlich-Nowoisky J., Elbert W., Andreae M.O., Pöschl U. (2012). Primary biological aerosol particles in the atmosphere: A review. Tellus B Chem. Phys. Meteorol..

[5] Violaki K., Nenes A., Tsagkaraki M., Paglione M., Jacquet S., Sempéré R., Panagiotopoulos C. (2021). Bioaerosols and dust are the dominant sources of organic P in atmospheric particles. NPJ Clim. Atmos. Sci..

[6] Mosalaei S., Amiri H., Rafiee A., Abbasi A., Baghani A.N., Hoseini M. (2021). Assessment of fungal bioaerosols and particulate matter characteristics in indoor and outdoor air of veterinary clinics. J. Environ. Health Sci. Eng..

[7] Li M., Wang L., Qi W., Liu Y., Lin J. (2021). Challenges and Perspectives for Biosensing of Bioaerosol Containing Pathogenic Microorganisms. Micromachines.

[8] Taş N., de Jong A.E.E., Li Y., Trubl G., Xue Y., Dove N.C. (2022). Metagenomic tools in microbial ecology research. J. Microb. Ecol..

[9] Yuan C., Wang X., Pecoraro L. (2022). Environmental factors shaping the diversity and spatial-temporal distribution of indoor and outdoor culturable airborne fungal communities in Tianjin University campus, Tianjin, China. Front. Microbiol..

[10] Yooseph S., Andrews-Pfannkoch C., Tenney A., McQuaid J., Williamson S., Thiagarajan M., Brami D., Zeigler-Allen L., Hoffman J., Goll J.B. (2013). A metagenomic framework for the study of airborne microbial communities. PLoS ONE.

[11] Fang Z., Ouyang Z., Hu L., Wang X., Zheng H., Lin X. (2005). Diversity and dynamics of airborne fungi in Beijing during summer and winter seasons. Sci. Total Environ..

[12] Lee Y.G., Lee P.H., Choi S.M., An M.H., Jang A.S. (2021). Effects of air pollutants on airway diseases. Int. J. Environ. Res. Public Health.

[13] Custovic A., Simpson A. (2012). The role of inhalant allergens in allergic airways disease. J. Investig. Allergol. Clin. Immunol..

[14] Zukiewicz-Sobczak W.A. (2013). The role of fungi in allergic diseases. Postep. Dermatol. Alergol..

[15] Zhang Z., Zhao M., Zhang Y., Feng Y. (2023). How does urbanization affect public health? New evidence from 175 countries worldwide. Front. Public Health.

[16] Calderón-Ezquerro M.C., Serrano-Silva N., Brunner-Mendoza C. (2020). Metagenomic characterisation of bioaerosols during the dry season in Mexico City. Aerobiologia.

[17] Calderón-Ezquerro M.C., Serrano-Silva N., Brunner-Mendoza C. (2021). Aerobiological study of bacterial and fungal community composition in the atmosphere of Mexico City throughout an annual cycle. Environ. Pollut..

[18] Calderón-Ezquerro M.C., Gómez-Acata E.S., Brunner-Mendoza C. (2022). Airborne bacteria associated with particulate matter from a highly urbanised metropolis: A potential risk to the population’s health. Front. Environ. Sci. Eng..

[19] White T.J., Bruns T., Lee S., Taylor J., Innis M.A., Gelfand D.H., Sninsky J.J., White T.J. (1990). Amplification and direct sequencing of fungal ribosomal RNA genes for phylogenetics. PCR Protocols: A Guide to Methods and Applications.

[20] Magoč T., Salzberg S.L. (2011). FLASH: Fast length adjustment of short reads to improve genome assemblies. Bioinformatics.

[21] Su X., Pan W., Song B., Xu J., Ning K. (2014). Parallel-META 2.0: Enhanced metagenomic data analysis with functional annotation, high performance computing and advanced visualization. PLoS ONE.

[22] Bengtsson-Palme J., Richardson R.T., Meola M., Wurzbacher C., Tremblay D.E., Thorell K., Kanger K., Eriksson K.M., Bilodeau G.J., Johnson R.M. (2018). Metaxa2 Database Builder: Enabling taxonomic identification from metagenomic or metabarcoding data using any genetic marker. Bioinformatics.

[23] Escobar-Zepeda A., Godoy-Lozano E.E., Raggi L., Segovia L., Merino E., Gutiérrez-Rios R.M., Juarez K., Licea-Navarro A.F., Pardo-Lopez L., Sanchez-Flores A. (2018). Analysis of sequencing strategies and tools for taxonomic annotation: Defining standards for progressive metagenomics. Sci. Rep..

[24] R Core Team (2013). R: A Language and Environment for Statistical Computing.

[25] McMurdie P.J., Holmes S. (2013). phyloseq: An R package for reproducible interactive analysis and graphics of microbiome census data. PLoS ONE.

[26] Kandlikar G.S., Gold Z.J., Cowen M.C., Meyer R.S., Freise A.C., Kraft N.J.B., Moberg-Parker J., Sprague J., Kushner D.J., Curd E.E. (2018). ranacapa: An R package and Shiny web app to explore environmental DNA data with exploratory statistics and interactive visualizations. F1000Research.

[27] Kassambara A. (2023). ggpubr: ‘ggplot2’ Based Publication Ready Plots. R Package Version 0.6.0. https://CRAN.R-project.org/package=ggpubr.

[28] Dray S., Dufour A.B., Chessel D. (2007). The ade4 package-II: Two-table and K-table methods. R News.

[29] Wickham H., Wickham H. (2016). Getting Started with ggplot2. ggplot2: Elegant Graphics for Data Analysis.

[30] Ggvenn. https://github.com/NicolasH2/ggvenn.

[31] Šantl-Temkiv T., Amato P., Casamayor E.O., Lee P.K., Pointing S.B. (2022). Microbial ecology of the atmosphere. FEMS Microbiol. Rev..

[32] Nageen Y., Asemoloye M.D., Põlme S., Wang X., Xu S., Ramteke P.W., Pecoraro L. (2021). Analysis of culturable airborne fungi in outdoor environments in Tianjin, China. BMC Microbiol..

[33] Van Rhijn N., Bromley M. (2021). The Consequences of Our Changing Environment on Life Threatening and Debilitating Fungal Diseases in Humans. J. Fungi.

[34] Van Rhijn N., Coleman J., Collier L., Moore C., Richardson M.D., Bright-Thomas R.J., Jones A.M. (2021). Meteorological factors influence the presence of fungi in the air; A 14-month surveillance study at an adult Cystic Fibrosis center. Front. Cell. Infect. Microbiol..

[35] Abdel Hameed A.A., Khoder M.I., Ibrahim Y.H., Saeed Y., Osman M.E., Ghanem S. (2009). Study on some factors affecting the concentration of indoor and outdoor airborne fungi at different sites in Cairo, Egypt. Indoor Built Environ..

[36] Jones E.G., Suetrong S., Sakayaroj J., Bahkali A.H., Abdel-Wahab M.A., Boekhout T., Pang K.L. (2015). Classification of marine Ascomycota, Basidiomycota, Blastocladiomycota and Chytridiomycota. Fungal Divers..

[37] Ortega Rosas C.I., Calderón-Ezquerro M.D.C., Gutiérrez-Ruacho O.G. (2020). Fungal spores and pollen are correlated with meteorological variables: Effects in human health at Hermosillo, Sonora, Mexico. Int. J. Environ. Health Res..

[38] Nageen Y., Wang X., Pecoraro L. (2023). Seasonal variation of airborne fungal diversity and community structure in urban outdoor environments in Tianjin, China. Front. Microbiol..

[39] Dye M.H., Vernon T.R. (2005). Air-borne mould spores. N. Z. J. Sci. Technol..

[40] Rosas I., Calderón C., Gutiérrez B., Mosiño P. (1986). Airborne fungi isolated from rain water collected in Mexico City. Contam. Ambient..

[41] Aira M.J., Rodríguez-Rajo F.J., Fernández-González M., Seijo C., Elvira-Rendueles B., Gutiérrez-Bustillo M., Muñoz-Rodríguez A.F. (2012). *Cladosporium* airborne spore incidence in the environmental quality of the Iberian Peninsula. Grana.

[42] Olsen Y., Skjøth C.A., Hertel O., Rasmussen K., Sigsgaard T., Gosewinkel U. (2020). Airborne *Cladosporium* and *Alternaria* spore concentrations through 26 years in Copenhagen, Denmark. Aerobiologia.

[43] Ballero M., Piu G., Ariu A. (2000). The impact of the botanical gardens on the aeroplankton of the city of Cagliari, Italy. Aerobiologia.

[44] Calderón-Ezquerro M.C., Martinez-Lopez B., Guerrero-Guerra C., López-Espinosa E.D., Cabos-Narvaez W.D. (2018). Behaviour of *Quercus* Pollen in the Air, Determination of Its Sources and Transport through the Atmosphere of Mexico City and Conurbated Areas. Int. J. Biometeorol..

[45] De Foy B., Clappier A., Molina L.T., Molina M.J. (2006). Distinct Wind Convergence Patterns in the Mexico City Basin Due to the Interaction of the Gap Winds with the Synoptic Flow. Atmos. Chem. Phys..

[46] Kirkland T.N., Fierer J. (2019). *Coccidioides immitis* and *C. posadasii*: A review of their biology, genomics, pathogenesis, and host immunity. Virulence.

[47] Priyamvada H., Singh R.K., Akila M., Ravikrishna R., Verma R.S., Gunthe S.S. (2017). Seasonal variation of the dominant allergenic fungal aerosols–One year study from southern Indian region. Sci. Rep..

[48] Nie C., Qiu Y., Pei T., Qin Y. (2024). Specific Sources Exert Influence on the Community Structures of Bioaerosols. Aerobiology.

[49] Morrissey C.O., Kim H.Y., Duong T.-M.N., Moran E., Alastruey-Izquierdo A., Denning D.W., Perfect J.R., Nucci M., Chakrabarti A., Rickerts V. (2024). *Aspergillus fumigatus*—A Systematic Review to Inform the World Health Organization Priority List of Fungal Pathogens. Med. Mycol..

[50] Egbuta M.A., Mwanza M., Babalola O.O. (2017). Health Risks Associated with Exposure to Filamentous Fungi. Int. J. Environ. Res. Public Health.

[51] He T., Jin L., Li X. (2023). On the Triad of Air PM Pollution, Pathogenic Bioaerosols, and Lower Respiratory Infection. Environ. Geochem. Health.

[52] Simon-Nobbe B., Denk U., Pöll V., Rid R., Breitenbach M. (2008). The spectrum of fungal allergy. Int. Arch. Allergy Immunol..

[53] Corzo-León D.E., Armstrong-James D., Denning D.W. (2015). Burden of serious fungal infections in Mexico. Mycoses.

[54] Abrego N., Crosier B., Somervuo P., Ivanova N., Abrahamyan A., Abdi A., Hämäläinen K., Junninen K., Maunula M., Purhonen J. (2020). Fungal communities decline with urbanization—More in air than in soil. ISME J..

[55] Liu L., Zhong Y., Ao S., Wu H. (2019). Exploring the Relevance of Green Space and Epidemic Diseases Based on Panel Data in China from 2007 to 2016. Int. J. Environ. Res. Public Health.

[56] Hughes A. (2010). Disturbance and Diversity: An Ecological Chicken and Egg Problem. Nat. Educ. Knowl..

