# Supplementary material for: Diversity and Composition of the Airborne Fungal Community in Mexico City with a Metagenomic Approach
Source: Microorganisms. 2024 Dec 19;12(12):2632. doi: 10.3390/microorganisms12122632 (PMC11678110; doi:10.3390/microorganisms12122632)
Supplement: Supplementary file 1 [file microorganisms-12-02632-s001.zip › Figure S2.html]

Javascript must be enabled to view this page.

magnitude
magnitudeUnassigned

Rainy\_Center
Rainy\_North
Rainy\_South

633457543263623805

633457543263623805

12

12

12

12

12

12

105294109328161899

41

41

41

41

1

4

820659283731

1113730371

1059707358

1

1

255133124

8815

247125109

770562211

35814

466429

689490168

7111

7111

51

51

221110

18109

411

542313

542313

336

421

47186

22724663

22324562

22

22

15145

31

221

1

1

332

3

532

2

13018117

26

1

1238

73

1

1071686

1

2

1

1

1

494034

494034

2676

11

2566

1

1

411

411

411

9825

9825

17

17

988

1

888

215323111613

30169

30169

30169

22

22

22

1

1

1

158188115

846648

796347

531

214216

1

16359

456

111

538051

538051

372937

568

568

272228

1

31

33

11

171923

1

1

11

1

511

1

11

31

142817821122

485572307

17168

17232

223010

2119146

856343

2

25034068

718130

232829

121

545

13

172120

287297221

12614192

155153124

635

12176165

412

232

926

1

10766145

21

10128

241

887

502797392

785823

1218848

61

276631291

3

211729

561934

241112

11

231111

32822

30817

5

2

482976

482976

432875

511

10928

314

314

7824

7824

211135100

20413494

1

131212

48122

667

12191

11

494917

2397

12

31122

201542

716

1

1

1

34

22

17210490

135

135

131

131

1709884

353440

848

1276036

470426331659

281418

1147

2

213

932

171011

171011

467626191641

1571515437

414148104

42910582

11

24199121

745

1652

322

1857745

2757476

3821

3821

15298

1

10271

1

417

308720671175

341116

40029396

1119923

11

20371373542

319141429

1

18514968

634

634

634

634

634

5397

5397

5397

5397

2

3397

562866

562866

562866

562665

2

562663

21

21

17101267573

1014033

21912

21912

1859

343

803121

803121

772115

3106

14291116450

14291116450

371

371

55

33

22

590274197

814

13711989

625819

3215364

624321

828834243

777844

2311

731821

14272

641700176

314

314

17510067

2

2

2

1759867

1719362

341

1688961

455

454

1

51123

51123

51123

51123

13

13

13

13

13

260216208

260216208

16911186

16510982

1

20276

5127

883924

227

492938

424

42

4

293241

122

11

21

242115

202114

1

31

4923

2712

2210

1

1

1

11

11

11

484970

6910

339

34

21

424060

13522

293538

132410

132410

132410

914

914

914

914

914

393

393

393

293

263

1

2

1

1

103516061008

310

23

23

23

17

17

17

10321606998

10321606998

1

1

484883

222

464681

222829

222829

8858150

8858150

5652107

282294

242713

43

687348

22

1

152118

1

515028

543

222

321

7441343578

7441343578

8695986126150327

702551516845

282318

282318

282318

682649836548

10891079252

21118880

878891172

373725334192

915441584

180514691823

113118371

393362

7784001192

7464134

13826

175611621905

175511601903

122

131315

697

115

632

1

19171

19171

767155

262529

504626

136108128

221

1018

946677

303942

171145279

52434

52434

166121245

9474213

724732

199146119

16197

16197

16197

957261

957261

957261

885551

885551

745049

1452

456136683149

395631042144

3221

3221

395331022123

395331022123

536500770

12

12

432627

432627

236

236

7785325

4532278

161

314746

3

3

128

11

117

10983128

421

9977119

324

324

909464

909463

1

302591

23

112

23

15938

3

1

101342

1

616

616

211120

211120

15616989

22

34

339

13314953

10514

5213

4

1

1

1

1

6964235

5458201

52

12

212

5046190

255

15634

15634

122113521582

229258294

229258294

5154131

178204163

199233441

199233441

222327

4653172

11924

11

599

114138209

640688514

640688514

640688514

153173333

211923

211923

132154310

132154310

121611153325295

209174400

208172396

208172396

124

124

151211723142

99123487

6963375

3060112

141310492655

141310492655

535432176

535432176

29524092

24019284

1

1

1

94510824334

94410814334

94410814334

11

11

304206384

149112264

149112264

15594120

13983101

161119

5585261255

386372702

372354686

141816

172154553

170146550

283

23

23

23

454842115718

239

239

31

31

535

535

7171103

7171103

3

3

884

884

2

2

382

382

8487165

8487165

8769151

7964147

854

336631164211

113139105

557507457

269224623641

344

144

731741742

33

585597464

143141278

967

967

17394314

17394314

334

21

313

5662841821

5232401764

5232401764

131618

10112

3516

302839

421

262638

452318409

443449

423044

245

408284360

408284360

11133

11133

11133

251731147650

250230997609

10185230

240130147379

151541

151541

11

11

11

311721371738

142314

142314

142314

907595672

907595672

125105117

10720

627370383

352

11

1

12896109

3114

111025

219615191052

20271412877

20271412877

169107175

164107162

513

286220671477

286220671477

286220671477

286220671477

5713

5713

434

434

149

149

4049100

273074

273074

27

272867

131926

131926

131926

9683222

9683222

9583221

9583221

11

11

1

1

1

1

438654991574487

657651473

268191104

425

18010559

848440

278358239

151721

263341218

827759

827759

292571

292571

293693371344624

429547176088

121116

122312471658

342357

162220922399

174153292

2

908034

112410951609

141623

250742899638536

12612574

201324941780

229352637736682

309195319

309195319

321

2

1

311

4

31

2

1

22

2

823

1

14511

28108

7413

121

13

11

6312

3

1

121

213

5210

1

20935

631

122

2

1

15

11

32

1

2

181032

324

289

1165

1

91310

12

322

111

19822

11

233

1

22

2

151110

665

71

331

322121

5

1

11

1

2

11

4

12

332

3112

161

3

52

1

1

459

147

1

211

1

11

1

31

1

11

18

879

111

5

11

545332

545332

413927

13145

11769972148

252

252

11749922146

126100199

1

13410

13415

12

2116

171936

7676431239

26

1

631315

131626

100115488

593

389

435

6310

3114

121913

233340

135313911776

8106

25

656

131860

131860

298354327

242

474284

317

249305223

1

494571741

5185212

1

329380484

11410545

315320

264515

224

231

1

12

683333

452521

23812

197133366

197133366

895

1

795

846

846

696

241

11

354

31

31

206177193

206177193

11128

1

974

244

275

55

22

240197160

240197160

647353

654451

623

1057853

441038

31

31

28523

28523

414

414

947

947

3

3

12491188995

587442468

11

1

1563

14720

537

201916

537

515

21

132

411

122

272134

2

19314571

9270114

263513

122

1

312136

621

61010

361239

114

76

352626

4611

754

422732

31

23

1

8877134

112511

271339

9910

11346

1

25

302522

570662377

131865

557644312

4716

4716

254131021695

350352196

31

5103

2285

10413

22

170195109

17147

191610

17211

14118

1288

622

151110

41669

9511939

12

9411739

645707256

11211040

533597216

161417

1357

49

351

112011891046

1

1

11

1

7311265

551

9212

695

1

1

11

3

1

8815

1

132

1

1

1

13

380415336

12

1578

3

11

7226

211

411

12

532

222

345

253623

2

141

272416

10224

111

1

12

11

133

1

14838

1

1310

313

511

341733

2

441

51

11

1

1

1

421

1

1

1

11

214

1

1

122

7

162

1

11

131211

352423

395

225

346

713

1083

2

1

12116

3

12

11

1

21

1

231

124

11

597

322

524

1196

1

522

12

162

11

1032

285

142

1

7117

11

28218

34

274321

34

9711

235

1

212

221

1

47

11

1

1

12

326

1

857

110

11

11

9

1

1

132

11

1

17922

1

14145

5

3112

4106

1

252514

21

21

528

554

201035

1

16187

3

344

11

311228

1

1

15

44

649

1

1

81413

22

1

2

1

1

11

112

321

13

14

1

1

11

1

20104

1

21

122

1

72

2

2022108

2022108

29569933

29569933

2626143

589

367

222

332

1

232

2

2

759

3

21

1

3

1

1

216

118123

118123

3203121350

1311821178

1311821178

189130172

776563

713274

413335

1

1

1

181324

24

24

111

111

15823

211

13722

24146

24146

1

1

1

511

13

21

1

1

11

1382

27137291

25034874

13625015

7105

333

233

47315

222

1235

1

12

303532

12

982

6126

6126

151211

232

658

4

341

394224

10105

10105

873

873

161711

161711

585

3

285

243237347

210223334

292716

951

11

1

3

441

14

4862224

41

228

547929

43

6511

181921

28311

471

2

1

111

331413

30136

317

9911169211

9911169211

746

9511132197

33338

318205169

9510676

9510676

2239993

232024

2007969

721567389

721567389

721567389

3640524919293

166113228

166113228

119118456000

406987

111917345868

23216

92139

63510397245

5638626921

33115177

1

3862147

29

29

100712161013

2

434

982

6815360

235

443418

1

6

973788

1

21

221

1

2166

734

24

639

9119

31

272117

141819

1

231

3

2

82112115

8919

32

32771

3

193614

11

441

2

3137

56

3123

1

1

5101

1

814071

252235

134168309

11

14416

1

245343133

1

252116

15711

12

12

70171336

4513666

2535270

31

31

5688624459

11

228

234

2

5638574444

243200173

455043

455043

191719

16173

316

22

22

1133

1133

413

413

24392

24392

13686102

13686102

221

221

19126

19126

19126

170170425

170170425

2

2

170168425

170168425

328315297

707787

707787

707787

14139

14139

14139

244225201

244225201

41

9922

333221

1

151813

111

899071

34

14138

342432

242218

261

1275

334

295227172

1207764

1207764

1207764

543438

543438

22

412

52

433134

23

23

1

22

271119

14411

14411

1378

1378

606725

161712

161712

445013

23215

21298

141

141

141

232316

2

2

2

2

1

1

1

1

113

113

1

1

241

1

24

3

3

21

21

2

2

12

2

1

1283

23

42

1

1

531

1

21

21

2

2

1

1

21

21

1

1

1

879

879

778

11

1516155726335

1516155726335

1462137718763

5770

3052823503

2562226053

11

264

10659669

7878018463

541807572

541807572

4273481052

4273481052

152106353

152106353

10254423

10254423

173188276

173188276

907074017019

14551141820

472236

472236

14081119784

13371084613

7135171

505655

1158

738

42

17337

31

22

11

2

13

21

1

6246

1

221840

1

4

12

1

13

1

2

1

11

6437

51

34

1

663451145731

663451145731

663451145731

353047

353047

353047

526

526

526

784

784

784

351314

341214

341214

11

1

1

365

365

365

474949

474949

474949

799982288

718911273

201324

91

688894249

13

817115

817115

461185310

461185310

461185310

461185310

19092177

311

26892132

2550120892640

1

1

1

1

2414118212227

2357117852072

2357117852072

3142

2320116462007

3412563

3016120

283

283

261636

11

251635

21

21

272035

272035

272035

97122

97122

97122

97122

4461

4242

4242

4242

219

219

14

25

61918

61918

61918

1

3617

3121

328

318

318

318

1

1

1

211

211

211

211

113234193

4715955

4715955

336

4415649

6675138

6675138

6675138

7538881073

679833902

679833902

10156683

341313

244

1174

4638

1151

322318

111

1

223

311

492315

391612

422

541

1

1

767161

525542

201519

41

453683143

441675142

1281

5638150

12614

825

22

623

449

1

31

28

11

4432136

55

11

44

12

12

2

2

24107

31

21106

2211

2211

12

12

1

1

32

32

12114

12114

11

11

733

432

31

454

454

454

2

3

224

1166

1166

1166

1166

368

3

3

3

365

365

15

26

3

3

3

3

32579401500

544930

544930

544930

544930

31818701460

30888041381

33

3

3

30858041378

382171237

17422

2238

1347

941142

21895111

1

2338516951

936679

936679

936679

142

142

142

142

21178

21178

21178

21178

192658

192658

192658

192658

152639

219

2

1110

1110

1110

13

13

13

17

17

17

889

535

535

535

535

535

352

352

352

352

242

11

2

2

2

2

2

922266

3

3

3

3

3

892266

333

1

1

1

11

11

11

11

11

11

3

3

3

1

1

1

21

21

21

21

861762

861762

861762

4

821762

527490433317459980

150411682002

150411682002

157223385

181121

181121

358

358

132203336

132203336

4420

4420

11

11

11

11

11

11

1133813869

3

1

2

777520361

698450326

311

756834

1

1

353293508

346287506

32

442

17795672

12612

12612

20813

1

61

567

1

23

63

7242585

433

6839582

733962

733962

151068

151068

141060

18

21257

18235

18235

322

1

2

2

2

1

1

1

1

1

265102758541318

251413

251413

251413

251413

332

332

332

332

5326895

5326895

3372250

3372250

33323

1

32323

1624322

1011

2

722

1433919

1

32

32

32

32

209211605

5717

1

1

3416

1

3

111

1

11

1

3

1

1

1

1

21

21

591

13

1

11

1

461

461

5886215

1

1

3237135

3237135

3

3

111

111

1

1

254161

2

1

920

3116

61019

416

121

558

2

311

3

3

1

1

16

16

381770

521

521

331569

331569

122032

135

11

124

51516

51516

3211

329

2

3

3

1

1

1

8772266

8771266

1

5121222

354736

1

21

115

1

1

34

34

3

1

3

9534791916

284132379

3113143

361

287142

11857107

11857107

10352116

9846109

11

457

321013

321013

666657

65

65

606157

11

12

32

12916

16228

833

192527

97631014

241525

2

3211

31

2

1488

113

1

1

2

1

1517

1517

1

1

412738

18817

231819

12

12

12

4

4

62512

11

2

52509

2410406

2

1177

23

3

3221

2

65

3

766

2

2

12

182

1

1

13

13

422

31

31

121

1

12

502216464

432878

14554

2813

271511

413547

413547

321729

23818

9911

1

1

386135310

462770

2

33

324108230

77

4

12105

12105

141

141

714

714

45

45

247372678038654

237802580537345

211

211

639

221

418

2

2

248111

248111

6955326011802

313

712

4116123

1049

551171820

43

1915

11210

251135

8

368

12879386

19878763144

23

111

1

5334

11

14511

103

26

14640

13317

212

8128149

124

422147729

21

692576

1

121

8108

36

2213

1237

457

1

7311

13964141

324

757

14

500177577

11614

12

1

311

666

254

1185

2312

215

746

792983

1

391237

1

635

1

28920

4081991382

2722121

473941

31

3

135

2

624

1145

11

14818

12

19717

5410

13

2

161212

632

12680129

115

15

1

4118

2

13106

461979

9130

411955

11

1

14

2

7518

4812

2

1

28

210

75

131013

862375

22

1

121

40139141

393335

1428

231421

4728

391720

1

22

1079

9231172

232628

2

1

426

1

11117

5112

9246264

115

536

721183

162103484

1

19616

12

15536

22

253130

321758

12148205

413

111

609302847

1

21

2

429

229129495

1

639

1631130

1631130

167752242925392

18687260

7924

232356

445446

21114

498

52

174832

1

397378959

244

11

35

1

4948120

22097445

12

110

212

1053

85031563411726

214727

3352109

12714

212133

161114322

747

743

124

12

12

1

1

319183

16

17

1

1

31

121

20745

3

544

1

31

552177

11

1

555

54

1

12

1

3418163

784975685

10113

12

333

232

1

21

445830726702

1

11410

834

1

21

249462260

1578997

2

1

266319810

1057

1

4910

442

1108

111

1

661255

71716

2

223

6132127

2

2167

1

23

3513041362

326

22

1

2

922

22

112619

1

2

10519

34

15

51

631

927480

16109

1

1

1

11

626

132728

10147

515

14747189

522

2

24

2

361625

1

842

14638

10815

21424

21424

21424

8869141175

88110146

88110146

232624

53

24

14

11

1

678

11127

7747781005

142

1

1156441

31634

171535

810

1426

2

12

12

122

1

51111

285352

4827163

5955119

249

6450140

37

3112

241943

21

1055031

1

11

1

12

1

131917

11

712

7010870

3

150187173

586

12505

6223

34

1

1

6947110

372157

1

352156

2

11117

4

1

717

212536

212536

61

61

2

2

41

3

11

361227

361227

325

325

1859

1859

1

1

48

48

722

521

21

423

423

459735372397342828

218129224

423566

12620

12620

534

534

13164

13164

6419

6419

11

11

5618

5618

17694158

17694158

17694158

502441

502441

371629

371629

13812

1

973

319

66

66

66

66

7611

766

766

766

5

5

5

238242224684207942

196382174

164358119

150331115

14274

171340

2

171338

5911

5911

1024

1024

1003746871

253187127

1

11

251

249181126

22

22

267161217

72

259158213

112

2

444138

1

877

121211

201219

410

23

23

181882

181882

1

1

12

12

3224

3224

51

51

836

836

1731

1731

62

62

7714

7714

855

625

23

1

1

1

1

1038083

1

613

9116

11

857

13

332

1

255

614243

553

217

562

373741

1

353237

253

524

524

13

13

372627

1

312224

543

1049577

1049577

11

11

231219

161016

723

11101

11101

125

125

5

5

19209

19207

2

13

13

251778

251778

906856909

15

15

1055

1055

2

2

648457

112210

536247

146127133

1

61

138126129

1

112

22

22

401518

401518

120111119

120110119

1

12

1

2

41

1

1

21

111118

111118

515490

515490

21

21

18353

18353

12

12

13813588

13813588

218219270

218219270

505024

505024

53

53

1087

1

1086

191919

191919

22

22

22

1196335

1196335

865026

1

777

11

1821

631

235949222584205893

213156193

204145183

1

91010

234132218482203932

352235

11

2

142

192122

1258

11

11

1

1

131

9207

232994217376202995

202157175

432

809808621

12

435859

2072635252

2072635252

341

341

1

1

139413071514

876

1

394231

129311991406

545870

1

191727

5149

5149

14213

14213

15

15

433131

33

33

681

681

251126

251126

994

994

55

51

51

1

1

3

3

452401444

19126

19126

19126

7543

1221

1221

6322

3

322

3

355266310

152446

152446

1333

1333

524

524

989766

989766

11

11

411

411

18118

18118

875

875

11

11

11

11

361845

361845

15712

15712

1

1

815820

815820

658

658

251414

251414

292659

292659

11

11

5

5

5510764

5510764

5510764

161121

161121

161121

313929

313929

313929

313929

19839082045

2

2

2

6641267

6641267

221

13

1

333

6231

1

11

1

51

626

1

21

97196

1

3

1

1

281220

24

22

67249

67249

6249

42

1

18752195

18752195

27

18552188

251714

251714

1

1

15109

541

523

657553

657553

657553

15717211467

51

51

211

211

61429

124

51217

1

1

6

6126

6126

25495300

1

25495299

115

115

226137183

575

1

221130177

1352425

531417

7215

1

993

203429

203429

360140280

5

292114151

2717116

21

36712

101417

101417

431415

12

1

81

1

2052

10612

21

161

11

3

21

172

172

484228574

421199306

341010

551

184250

2

6105

1

1

1

1

1

1

332365370

313693

313693

313693

13512192

13311761

13311761

2431

2431

166208185

152118

91813

635

777746

23

373929

1

1

754

1073

71

1

43

14164

11

466778

466778

334

1

324

254039

254039

321720

662

662

662

16814

16814

311

1

11

1

1

1

1

776

15

1034

1

1

1024

1024

413

413

413

413

1

1

1

1

6306482511755

55714

55714

12

55612

5189382610845

10456991025

10456991025

163122141

163122141

398130059679

1

201010

396129959667

1

901709694

179101202

13081156

492046

722608492

722608492

161283202

31127

31127

123250189

123250189

41

41

7175

7175

212012140922119827

36

36

36

527663584810

527663584810

411948453465

115715131345

664515407335341

1147819824

1147819824

400127166

387122155

13511

323028492573

25109

352

11

22816388

3

16729688

280279267

662700529

12

22049177

704427

31

9143

1

725834

1

207164129

371128

135310480

170145101

91143

929587564

365927453300

543959

365257653

965136

1

979

413220140

1

425345374

362119

290171189

12

112

211177114

165713541630

1

10110072

1024735

984735

4

12

12

557174677527930

21126

48196

10137

12

843

11

1432

574545

13821

2

11

1

13

15101

1887

16102

406713335321186

36136

1384

1

1398

1

163126122

6116

4195

1

27202373993

11

17287134

14133

12

3496

16911760

1

553

1

1

2

35157

111

311

1139616407

59277

726922

1002997014836

22

68517

1

2

11

14163

2023786

2023786

402913

402913

1948641408

1948641408

544

544

361833

261

261

341232

341232

329394880

2

2

141511

141511

1219

1219

223

223

2

2

11

11

61514

61514

1167670

1167670

1

1

13

13

133

133

32

32

1

1

713

713

1

1

1395

1395

21

21

211

211

696

696

1252

1252

127240743

1

26629

1

281925

73214687

1

1

1

115

115

3419

1

1

3415

3415

3

3

305261200

422

422

211

211

545142

545142

1

1

4

4

32

32

182516

283

161713

936562

936562

13011177

13011177

181310

1

1

34

34

14136

14136

168161211

144134126

144134126

28

28

221985

221985

211

211

1

21

2238

2238

2238

2107990960

1835793881

1835793881

3649

3649

303420

303420

13111

991

42

42

42

1814

1814

924

924

37

37

13812637

231

35608

1006328

1

21104

21104

2

2

1

1

1328455

379

379

131

131

966829

966829

1

1

2

2

17213

17213

1333

1333

6154311591

38835

43

34832

5654091426

182188808

432840

13452190

206141388

524

21

3

23

712126

712126

11071121

11071121

11071121

341312363

27544

27544

123182238

120176236

362

1558471

1558471

364110

364110

725575809457

13134

13134

539556787

539556787

326364257

21

324364256

4911

499

1

1

191513

191513

14102

14102

996

966

3

862

41

452

120611282538

1

120311202532

376

1

1

131226

131226

24169

24169

32030492

32030492

476051375710

336937724508

783

138413571199

290141257

290141257

1

1967

12653127

442144

1006179

125810381295

11

11

311110

311110

621507566

5034167

571473399

20421

20421

45

45

586511691

586511691

1

1

18348501478

18288491477

18288491477

2

2

411

411

251814

251814

251814

283318611721

424232

424232

277317981657

1

277217981657

162026

162026

216

216

16151

22

22

791

791

74

74

291612

261512

261512

31

31

638

1

1

21

21

427

16

221

1

260225225

453625

453625

12211592

172810

685460

373322

9144

9144

8155104

8155104

35

35

182626421856

171525481714

114118551129

378458390

196235195

11194142

11194142

1369298574898

2

2

62268

62268

1

1

10075155

10075155

13751002455

1366996446

969

148176109

148176109

261122451268

261122451268

21541412439

21541412439

1

1

534

534

3312169

3312169

365203108

8112

357192106

997952

997952

533978

533978

688281

5

638281

291034

21

27934

645650336

569591295

765941

8219

5168

351

574337171513

574337171513

31

1

21

1176034

1176034

513243

513243

19123

1973

4

1

1

1

1

1065525339053706

87110105

16521

7110584

312

312

1

1

221160130

824

213158126

344

344

1

1

31

31

5

3

2

11

11

268415481674

268415481674

995

995

1032795140851621

1032505138651615

27226

2

15086112

721757

231614

555341

71010

48

652

11

332

332

832

711

1

21

332717

451

292216

611420

1

1

1769

31

1

2969

5

1

2

211

1

1

1

9554169

272544

272544

313

313

151063

141063

1

26650

25650

1

1081

1

981

1448

316

711

421

412150

154

1

144

1

1

391642

391642

4

4

752

12

12

632

632

733168

733168

191018

1126

431944

5969117

5969117

5064103

5064103

9514

9514

256772251160271

12127

3

3

3

9127

9127

1

1

9116

5493520620526

11627

11627

11627

485477351

483474350

361237

13176

434445307

231

231

1358783

947071

1176

836365

401712

401712

1

1

142236362

142236362

4123

1810

1

71438

3815

5069127

8310

33144

6910

599385

4113949121

3252428855

3

186105328

1391378524

86152266

131844

73134222

433430850

165215441

165215441

268215408

268215408

1

1

26

26

26

1

1

1

10207652401

47112

47112

6414842211

6414842211

10411891

10411891

1

1

12196

12196

21613291

906917

11117

1155267

650229503

1

1

1118144

31

511139

123

242

212

212

521150290

521150290

21

21

873050

9162

751232

3112

14

11

11

15

15

217

217

111

111

873

873

1113

1113

36

36

593

531

531

62

21

41

5163

5163

5163

98510283526

152

152

2523121807

4220

47111985

159157719

304261

1221

1

403540

403540

1812113

1812113

3938124

3938124

417

417

9583231

9583231

131012

4

1

11

21

212

622

253

7211353

589547

14186

615276

615276

16369

14369

2

457

457

370359985

102389

7956242

12358

256273589

1347

494515515

176168209

311627

3

11

211

1

343458

12

11

1

72

565

10499109

1042

1042

171142200

171142200

19238

19238

72812

72812

4131

4131

1194

1194

9612879

11

708736

9123

162939

71510142774

4206031359

23308

238430576

10189700

503845

81630

154217513

154217513

1

1

3431290

13

3028285

2

21

21

106163612

2

1317

315167

74107528

21612

21612

25

25

1

1

142

31

11

1

1614

3

1611

11

11

1

1

162827158073

11

11

11

942

942

942

153526588014

153526588014

153526588014

845256

12

11

1

21

21

44

44

921

921

1

1

3411

149

1

12

11

11

324

324

32203

32203

1

1

1

1

131013

1077

336

332

332

16

16

821

821

728

728

1

1

1

1

99110104

99110104

1579

1579

1

1

6415

5412

13

235

235

181219

13

1

56

19

1247

26

26

16

16

496332

1

1

6

162010

264222

5111

5111

417

12

315

8185451024

1681

221

221

146

1

126

1

121

12

12

1

1

2

1

1

1

1

221716

221716

1258

2

8128

414043

181830

181830

1

1

12

1

2

1

1

2

2

829

829

11172

32

12

1

23

21

2

29

2

2

214

214

212

2

6931103

2077

1967

11

211392

211392

27114

27114

1

1

221924

1

1

221923

221823

1

81

1

1

21

1

11

1

1

4

4

615413818

614413818

410

132

31439

1

331113

21

31215

5

2

4112

17410685

312960

512

1

1

2

1

31

312

1

312

1

4

1167657

12

1

202728

21

311

14825

1

74

61

3

156

1031

12

13

11

122

133

1008320

1

1

111

431222

1

1

3

5

1

1

313

11

1

1

23

23

16127

16127

1163

432

132

32

32

32

342399372

342399372

1

1

1

1

13

13

1512

1512

352824

352824

573

573

422

422

1

1

154171217

154171217

2

2

7122

7122

119162123

119162123

1941372337

1941372337

1941372337

2523202

2128

100651873

313178

3617153

3

450250773694

202187292

125138189

204645

221729

13

6768103

46

1263

222974

143

547

1

12742

192

2520

422

1

12

2

2

352

1

342

231

231

461024

46924

1

532

532

532

108104112

1

1

272629

132

1

171824

1

843

2

2

241932

171425

212

545

299

299

24911

24911

8129

332

597

81512

81512

131310

131310

314037801882

1

1

10137

294

843

41

41

795164

281622

513542

1233

1

122

1011

1

1

201115

201115

35

35

1721

1721

258432801112

29164

313615

971

72

539497213

875074

152

311104865

1008991525

6810

333820

51

504556165

1

13109

921

345

707285

676984

331

763

533

23

585

585

1046

414

632

43

43

362

142

22

1

1

1

1

198203321

19941

179194280

546

3

14

312

1

3653161

2949140

31

4420

13610

13610

201733

201733

521

521

414

11

2

2

3

182420

182420

112

1

1

2

447

1

223

223

442

442

215

215

148177168

234

12

11

121

4814

2413

241

111210

1093

137

532

532

662

662

1

1

1214

1214

388

387

1

513

513

43

3

4

223

213

1

372

1

362

122

122

12

12

99121101

385653

616548

495330701

812

82

1

11

1

1

511

51

1

152

1

142

478321693

11

124

1

1

13

231

112

1

1

1

1112

121

2

332

278140437

1

2

4113

1

131

1

1

272319

1

142054

4516

2

241

542

131

1

1

21

1025

451

661

1

13

1

14811

421

1

117

1210

11

685347

9

11

224

21

3

1

2

141

1

615

148

312

1

1

1

21

51215

11

1

1

24

2

4

335

335

265

12

145

643465

6116

1

2

22

2

51

323

1041

1041

14739

14739

1325

1325

14

13

1

17910

17910

1

1

3

3

486

486

486

331442451

2112

2112

160167249

112

241366

495539

231414

187

201633

132116

183

1

123

272965

411

411

1

1

211626

211626

137252155

4106

968

171349

162434

5514447

23459

1

1392

721

721

3

3

2

1

1

13

13

1512186

1512186

1512175

1512175

11

11

2842821385

141817

131311

131311

156

156

312

312

211

11

1

1

1

2412541358

36

36

3440261

1326108

7531

16

1

645

12

11

110

140

11

2112

2134

11

4454632

2

1

2821144

1631484

21

102112133

515

496172

485056

12685

12685

25

25

3633

3633

3525119

3525119

217

217

424

31

1

113

4563

4563

2385

2154

12

2052

1

1

23

23

213

213

213

335

335

335

335

741

741

741

4

341

14814

14814

5

5

1056

1056

22

22

411

411

4838329

4838329

4838329

3513

214

1

1

7714

1

425

1

1811251

5518

8618

2

2

7651191947

7651191947

7651191947

136270134

458756

516754689

688068

561

561

561

561

6398493013018

6398493013018

6398493013018

114102151

9147148

8421

6294481542

2735180

146147277

140138348

12197032243

77610332291

377316602

239615864613

2

412

471368600

199152182

119

119

119

99103111

612

612

3

3

112

12

1

292

292

336

1

236

656486

656486

18237

18237

423

423

632

32

32

312

312

934560

934560

611524

431

1

52

3

2

13

3

531

22

622

21

1

25

1

485

483016788053

414211002663

414110982662

350100145

1

37909982517

121

11

11

251528

251528

161411

8116

11

231

231

231

24822

73

73

1

1

13518

13518

43

43

5925035242

1

1

137

137

1851221092

1851221091

1

11

1

1

11

1

1

41267

41046

221

8219

8219

3102682779

1

5238342

1

118

3623358

2318

6253708

1

1811175

17493

181225

1

1317152

90105889

84106

84106

35

25

1

5053821

24

5051817

2915

1

3

1615

5415

5415

1721311

218

3369

1

1313178

1345

112

11

2

454997

2

2

31

31

2531

2531

152732

72227

524

11

221

22

22

211632

2

14628

5104

1161512650

1161512650

1161512650

36

36

1024

7

324

11

11

636467

636467

6155129

6155129

1

1

1

1

3

3

17728

17728

21

21

7214

7214

11421

11421

682176205

682176205

656

241

415

1

1

961

961

992219

992219

142148110

142148110

3

3

481238

481238

42

42

42

42

42

1

1

1

1

1

21

21

21

21

21

624347686

211226

211226

211226

211226

603335660

603335660

603335660

409166568

13

19316692

356179199

965613

965613

821

821

885412

885412

936642

393

1

1

363

363

1

1

1

1

905739

551

551

372318

372318

32

32

452918

452918

1

1

1

1

16161

16160

13160

13160

3

3

1

1

1

431

431

431

431

1284

1

1

1

1283

1233

912

321

5

5

12

12

12

12

812

612

612

612

2

1

1

1

1

552740

2

2

2

844

742

311

1

4

2

1

11

11

1

1

341

341

341

2

2

2

21

21

21

391732

852

12

75

311230

311230

12

12

12

21

21

21

21

1

1

1

1

2

2

2

2

35511

35511

83

83

921

911

1

122

122

1

1

52

52

1

1

1

1

3

3

5

2

3

41

41

2646

2646

2646

2646

7417

7417

316

316

441

441

529

529

529

229

229

2

2

1

1

474777

474777

424476

424476

343562

22

126

558

531

531

531

806474

806474

636161

31

31

181913

171211

172

71621

2613

5108

12

12

555

555

291921

261821

31

17313

111

111

16212

16212

10638809111407

95101313

4154234

184

184

232579

212073

256

142059

212

2

71139

242

1412

22

3192

3192

133051

13

1

2

1

263

231

32

5719

5719

2518

2518

27

27

261

121

14

1

1

11

11

11

11

411728

3162

2852

31

2

2

36

34

2

71118

71118

288165181

15578103

15578103

15578103

1338778

1

1

424429

424429

332015

332015

572334

572334

131116

131116

131116

131116

9262748410354

141

141

1

131

714741

403424

403424

6

6

42

42

887

52

367

2

2

1158

1158

957483

957483

957483

124164133

487721

487721

1

1

10712

10712

101215

101215

41024

121

3823

1

1

2

2

1178

1178

491

491

11

11

101324

22

81322

242925

4

376

11

162119

8116

1

1

8106

543

363

424343978172

2

2

422843898163

12

422543858158

333

1

1

1464

1464

122

122

3324297

3021296

1

3020296

331

331

459426861569

526793

526692

11

866

441

425

51115

51115

11

11

2

2

3115493

3115493

1

1

814

814

27714733

27113820

6913

40392269686

566212220

76294

28001713259

456

481245166

1126531

385439

385439

252

251

1

544064

544064

301821

301821

364191

364191

412

11

311

1

1

5916

5916

1

1

11

11

2

2

767144

221511

683

1678

394532

394532

1581

1581

3

3

499

499

499

371836

371836

271211

271211

7322

7322

333

213

12

943312507

943312507

1417663

1417663

2245694

2245694

1

1

2594657

2594657

15567148

43

14967137

13

15

14763116

14763116

16429

16429

1

1

1

1

1

74

74

74

74

74

1138407458

1138407458

1

1

1

1445536

541

541

232

1

22

11

1374833

14

1112122

21

2

1

1

20266

12

12

2

1

40235283

1253

11

1152

5

5

32318272

2

32118271

1

131

131

4

4

1

1

4968

1

2915

1

1

91

1

623

11

1

12210744

1079943

644

61

959439

1581

1581

935227

86

4

31

15

30114

1

311

722

33

6

1

2

82

1

2

513419

59

384

431715

414

4

14

921

921

921

2184324

811

811

95237

811

61

1

3

39123

51

1

91

1221

1341

1121814

1121814

312

11

1

11

1

162

162

71

61

1

2

683

212

12

11

1

1

1

1

37

21

6

1

20118

523

3

11

113

1014

83

211

321

21

11

1

24

24

2

2

1078932

3

3

2756

732

12

1

1912

2

1

1

2

2

415

215

1

1

1991

531

146

10113

982

121

1

11

11

30414

24313

1

591

1

11518

11

8318

21

1

1

1

1

1

1

823

823

823

823

823

823

5635841836

500543397

321419

321419

321419

311

1

2

426

21

1

2

3

412

111

1754

456527331

1

1

1

7137

7137

7137

583067

573067

841

2

1

217

3

432049

13

1

1

382478243

382478243

5131

2

377465240

4

4

4

969

728

728

241

1

1

131

11244

11244

11244

4143

711

13

13

13

13

63411439

63411438

32221295

32221295

32221295

3119139

1

1

3019139

3019139

4

4

4

1

1

1

1
