# Supplementary material for: Diversity and Composition of the Airborne Fungal Community in Mexico City with a Metagenomic Approach
Source: Microorganisms. 2024 Dec 19;12(12):2632. doi: 10.3390/microorganisms12122632 (PMC11678110; doi:10.3390/microorganisms12122632)
Supplement: Supplementary file 1 [file microorganisms-12-02632-s001.zip › Figure SI.html]

Javascript must be enabled to view this page.

magnitude
magnitudeUnassigned

Dry\_Center
Dry\_North
Dry\_South

9981149534621233856

9981149534621233856

100843128048226081

1153

1153

1153

1053

12

211

74

1

1

349273350

349273350

349273350

349273350

193152219

442

152117129

2216733357169524

615052099893

554765

283426

283426

271339

8410

19929

21687146026

21326835972

21326835972

23823

11

23722

132331

122231

11

1

1

1

360353438

332322319

332322319

2831119

6118

1

2120111

516323198

490284189

490284189

26399

24387

212

109152157

71131136

70128135

131

382121

382121

606707843

603707841

581694827

221314

32

32

174219541502

216

216

1058

1048

1

11815477

9712559

212918

2813

2813

464338

424236

412

140016091233

7821016724

2

32296

584564503

1

1

121

121

1015353

1015353

321

321

2

2

362727

362727

12

12

214843

214843

275818

275818

11388

162010

522786553

520786553

520786553

2

2

151

151

151

255

255

255

1

1

1

4110487

11

11

4110386

4110386

32

32

32

32

1243100511697

806770741

427549609

111540

101740

51692

233

202246172

87131147

110121115

1

1

3

3

473

333

14

191313

124

215

16104

22412291

1

22312291

1287925

796011

491914

42823110934

3714484

3714484

39121710450

38620610434

51116

9422

9422

9422

194247210

194247210

194247210

194247210

27124973

17717439

16016235

16016235

17124

74

1084

24268

24268

24268

704926

704926

33

13

86

482617

2

1396

9173111

396

396

22

374

261150

26946

26946

24

24

55

55

55

1

1

1

1638

1638

1638

41032

41032

41027

5

252

252

252

8114

1

1

11

1

1

552

552

1

1

15

1

13

1

1

1

27234

64

3

34

21194

21194

3460838862670

3460838862670

3345802150633

2183792504

92326

3

5618289093

1632133

4598997141

2082586031733

11536712037

11536712037

4716

4716

216

216

2610

2610

353515

101511

101511

9141

1110

23194

23194

23194

21

21

21

273428645250

112

112

112

433117

413017

235

382512

12

21

21

16910663

16910663

16910663

4702

4702

4702

211420164875

211420164875

211420164875

3411

3411

3411

391614269

624

624

385612265

375601230

101135

395

395

395

21

21

21

4135

282

282

1

1

153

153

360778302

254646187

4814

4814

250638173

250638173

7110777

116

116

12

12

446

446

126

126

3

3

344016

344016

573

573

235

121

13

1

1

2

2

7117

12

357

34

123014

4102

1

11

71911

497

357

11

3

352538

572

572

301836

542

241432

2

1

231186314

7447150

531

531

6944149

6944149

658593

658593

454

436243

2

161846

814555

814555

814555

11916

11916

535

6611

71361402078610

8592105

293533

37

293226

178

178

26

1

13

1

1

1

534464

1

1

1

2

159

1

1

14817

1

444

11

2

131

1327

182

11

12514

213

2

112

21

21

1

11

3546955762078

1412393030077

1254364528438

67120611

1

911651027

22612

22612

69313237102

41121277

63911786731

132492

2

188147162

1

14

5330

322

1

2

613

301836

11

17

212

501919

1

1

3

1

1

71

15418

42

171

1

18

736

311

372713

47

1

12112

214

4976378

4463368

51310

1182407524347

1181407324345

112

1

422

22

22

22

1

21

515

515

12

1

31

1

11

546870

212634

31

182534

232817

17228

669

996

996

1513

1513

322348453

568052

416839

151213

17

17

27341

27341

292916

113

12

1

2

211710

31

2

21

13

11

131010

514

883

13

191186374

5

111

142

2

127

211

2

118

2312

852

76

524

324

447

1

4710

1

11

2

12

2

11

53124

23

4

3

1

12

1

1

76

1

923

11

33

1

21

632

1

1

2

14

3

2

1

1

1

13

1

1

1

29

11

4

113

1

9110983

116

1

2

1

1

2

11

22

71

1

2

1

19

1

4

1

14

52

52

743437

7214

2

7212

673223

673223

401725

401725

4

301112

6613

1849

1

1

1428

1428

211

211

11

11

8167

8167

475

18

312

2765225364

2535085351

2535085351

231413

234

964

1255

41

41

41

194150376

193149376

5

72021

4

1

151244

11

131027

21

3

31

212

1

8576

123

41

1

2326

11388188

11

1

1

442326

1765

1765

71

71

1

1

152

152

11210

11210

666

646

2

232

121

111

282231

282231

282231

15811

2

2

14

1

13

21

21

12210

12210

209628489184

160423287623

203416

156922837596

151111

4925201561

865

11

128118445

201524

149113446

14

1822124

168241516

4046201

2740186

2740186

133

1

13

1

1

22

22

10112

10112

271616

343

1

32

211

23911

1

1316

1

2

1

371

1

1

1

31

132

132

217191575

1227

1226

1

22

22

202419

322

171815

42

331

331

332979

332979

6790406

1

322759

144

3458343

297

297

642213

45

601713

271021

271021

233529

152

152

223027

1

2

1

1

1

2

1

1

116

1

2

1

11

1

1

31

1

2

1

11

33

1

2

11

1

12

4

2

1

1

4

1

21

1

2

14186

14186

2

43

1

1

3

1

171

1

23

161

333525

192222

192222

73

1

11

462

541

1

12

11

1

1911

64

64

64

893

893

893

182057

182057

182053

182053

4

4

154150169

154150169

71122

71122

127124135

127124135

201512

201512

21

21

21

21

5386109

5386109

5386109

5386109

641

641

641

641

521

12

548428129644055

547918124844023

7164781873

7164781873

7164781873

13566245

7125

7125

523127

502826

231

7534193

7534193

1

1

539408070441905

539408070441905

23647

538738056941871

447127

6

2

2

2

4

4

4

1

1

1

1

1262

61

61

61

652

652

652

1

1

1

1

222427

122113

122113

112013

11

10314

10314

10314

11

11

11

11

9172

9172

9172

11

4152

41

18181323

18181323

18181323

18079267

101074

17069193

1256

2

3

153

18789104846530

969946413133

962646283097

498022451483

29551406441

451714

691295190

2029529

462241145

32

622189664

1716

1716

462523781582

23720

1190503199

118409

942378346

748307190

686

797606311

767517488

341213

4516

254

212

731336

17613

17613

56723

4

14212

38511

282141173

137

137

137

281138166

1477670

1477670

31

31

362418

292118

73

511

511

261626

3

1

224

822

1

211

11

846

10

33

2

1

642051

2

237

11

622

11

26525

1

63

21

1643

429

1447681456

26311097

26311097

1252

2155

2269990

41

1184571359

179

179

311110

27510

46

716333

1934

526029

1016

1016

21

21

1055494300

80821

836435239

1395140

924620

924620

924620

924620

726949752748

445156164

935

935

411

411

432152158

32710585

622741

432032

7332109

35815

31

1

21

913

51

21

311

848

21

352190

311986

1

31

14

334

334

401175189

661912

421

733

53147

21

335156177

214

333155173

511241551817

675390267

111

1

21516499

416216157

43810

266122170

668

111

511

21

31

36148

213100150

221811

311

11

11

11138

73

20281576808

18411372

203258172

13211

16

811717304

816480249

1

1

21212048561

9017931

301716

10271150275

32014791

50235495

927234

2

5812919

472019

472019

472019

717263246

105

6

12

1

1

13

707258246

55106

1434445

1

221

257106101

1

37168

1

2

803743

993618

23412

7311

418151177

344110143

1915

325109138

251512

251512

492622

11

2

20515

26194

22

985

985

985

512

512

512

321014

2667

2667

647

646

1

1046

1046

214

832

4

4

4

4

4

2424

2424

2424

2

2

424

424

23

23

23

23

23

2169871996

353

353

353

353

480191162

480191162

13414

1146

28

467187148

32

464187146

1554621701

1554621701

788358277

692411

1714972

473426

3146

470247162

916

916

735255409

30366183

9114

663712

249122134

1082966

535

112

423

1744

1744

1325980

612435

612435

472131

1434

713545

713545

683443

312

583228369

611

611

611

611

10453135

10453135

10453135

10453135

19109

19109

19109

19109

454164224

337137169

328137168

14912

1269

11

2008168

471322

9317

391530

799

91

1

81

11

11

1

1

1

1

1

1162654

1162654

1162654

9338863398

934

111

111

111

823

823

823

1

1

1

1

9248833393

9248833393

10481726

10481726

11643

6543

51

15727

9324

643

2724201

5431

2220170

5625721494

5625721494

7946152

7946152

100129637

232681

1

76103556

2618113

3432

12

14642

13

9634

561453319

476414197

476414197

191105110

1236

431

26927

121

249

793019

265

334

411

323218

54

4224

423017

1

422815

1

11

19525339

11

19425239

482631

261514

141013

814

341

341

1

1

1

1

24

24

11

11

11

11

18867

15864

15864

11857

47

33

33

33

472539

271920

1

1

674

12

572

21

21

12

12

1065

865

2

25

1

24

13

13

2

2

3

3

3

3

1

1

1

1

20619

31

2

1

1

17618

10314

734

16214

16214

16214

45

524

1

34

31

23796129

23796129

1556284

1506177

36911

564

662429

351828

442

43

517

31

13

14

221

221

221

2165

2165

1865

3

33925

23518

27

21511

822

612

21

225

225

261714

201112

1356

766

662

31

251

11

11360

11360

11360

11360

1

42

1

221

2

2

1155

895644824114981787

4620

4620

4620

4620

4620

9417

9417

9417

823

823

1214

1214

114

114

114

114

114

13064969913936

169187

169187

169187

169187

7794770110639

859961

111317

111317

386717

386717

14

14

351527

7315

281212

386041852813

1

1

636861

636861

21235158

20033754

12144

847

847

669670

648960

2710

112

1

111

753

753

343636

343636

1

1

31

2

11

101214

61111

413

585964

585964

454354

454354

4315

4315

2

2

421

421

332234792414

1

545313215

11627021482

12932034627

11

28238380

1

394410

18216

18216

11

11

335

335

763

763

763

14586148

28128

1

28118

622328

622328

37

37

22

22

22

22

161410

1

151410

10105

10105

1

1

1

1

8855

8855

1

1

222

222

151129

6323

986

15439121

22

22

15237121

15037117

24

1337

1337

1022

214

11

141311

141311

141311

334

234

12

222

1

1

342231847399

1086

1086

1

1

1

1

232

232

1

1

340831727391

340331697382

1

439

918372

211

211

411

411

452

452

21

21

15827

11

11312

3514

646840

646840

467815911110

467815911110

43119

43119

1

1

2454596370

2454596370

512231196

509228195

331

284100117

284100117

479191100

479191100

906462317

5

890458317

84

3

1174576

1174576

531833

531833

612642

612642

311

311

402426

271522

724

724

966

966

21

21

9711

9711

1394

1394

1394

4193291898

185106461

11257252

11257252

511

1

411

1416100

1216100

2

5432108

5029103

435

1851791138

6944455

221071

4734384

152166

152166

8499358

14611

3424

64218

413

4643262

11340

1715259

1715259

4944299

1

1

213

213

131

131

15715

618

42

465

1

843

843

7511

338

423

1

1

820252

820252

6414

112

5312

320314652302

320114622300

320114622300

320114622300

20158511640

421721

1144594639

232

232

232

12

22

4257440229221107

10227120407969

737598064504

112

112

945

121

222

1

52

31

1

21

22

22

966

223

12

623

670589601446

26

32

14311255

15

2789

1764

22054452482

21

1

4101

4112

37187

35406

1

1

8511025132

10134

1049426

815

1371

352311

31333046672

706434

143

143

32

21

1

1

6149129

5848102

3127

651

1

1

12

1

33

111

111

4845166

172599

1

302067

37166

37166

474

24

44

1

31

31

321617

321617

885

231

654

22

22

121114

121114

1

1

586381

454470

131911

8410

21

418

411

1491952496

1361902477

618

7411

778444

778444

11

11

13732459

582

13231657

445266670

915

915

511912

1865

33137

1

1

752

752

21

21

2

2

290186568

11593361

17593207

321

321

2

2

43

43

121

121

4210

15

12

113

11

123

123

392230

731

321929

211125

111

201024

41

41

221

221

687

341

346

211014

211014

211014

308298202

12114645

11

531

24106

572116

3411122

16915

16915

91034

91034

574641

11167

443031

23

1

1

181816

181816

353716

2

333716

291424

291424

221810

221810

1

1

433465

301741

337

1

201320

111

412

11

535

535

1

1

337

337

35

35

351

351

226

121

13

2

1149821753

11

11

6328

6328

468337247

451

21

23213781

485327

11525

194131

2

19119

1338372

22

22

143

143

345

345

4120120

4120120

9512

9512

583441324

13782124

482940

91

594529

1736516

12

21

271527

4102

653

41

11318781

1626

1626

2

2

1935

1935

3362241324

3282201312

1

1

1

825

1

161113666

6

1

2

2113

2

12

4215195

21

12

2

4

10114

8

122

2

298

641

1

218

32

11

214

2

2

13

1

2

272486

3

1

1

8678

314

1565

13945

2

2347

135

11

24

2

2

16

1

22

327

2

25

4623

123

11

11

324

222

12

43

2

1

1

1

1

1

1

1

11

11

12

1

2

14210

14210

14210

521570409

18216

1122

1794

160166105

21

158166104

484254

1

763

2

511

447

523

232333

347

289340231

527355

13

352

12

182021

651

162177138

465811

115

115

58

2

24

11

3

15918

211

211

313

313

9613

538

435

111

1

11

1574

1574

1574

1574

1987

1986

1986

654

1332

1

1

1

341176261

1

1

1

236109205

975442

1

965442

1333

1333

1983

1983

701917

701917

2313123

1

21

2112122

16

16

523

21

21

112

998

33

695

16

16

16

2

2

2

1036550

1036550

1

1

1

7103

112

1

43

431610

1

1

3

11

32

9143

211

924

1

1

1

333

11

21

9512

12

12

1

24

1

14791149371

116

73

73

43

41

2

604396

604396

402846

201550

2

2

2

334

1

1

11

11

212

212

11

11

779

1

1

1

1

669

669

222

222

222

92

3

3

62

3

32

151133

151133

26

6432

11

61

26778

1

1

19612

1

13592

61

1

1

1

1

2

2

4155

4155

1033

1033

521

512

1

1

1

72552772

16103

16103

13215610

1

1281478

392

57736159

57736159

1

1

1

620461138

620461138

31

2

1109937

211

1

1

3

312

14

23

21

1

221

1556

1

13

1661

3

1

18

70167

11

91414

632

4

2116

11

11

21

1

1

1

1

11

22222

42846

1

543511

1609214

41

39335

4

1

1712

1

1

1

2

31

2

112

11

11

43

392498

6

6

6

392492

14745

14745

13

13

36

36

16

16

17717

9

925

743

11

1

1

6614

23

3

1

4

616

1

1

251348743

7975135

29

28

1

21

21

21207

21207

21

21

5

5

12

12

31

31

4812

4812

3

3

3416

112

2314

12

12

1

1

216

216

10911

10911

262559

4715

221844

156267589

156267589

156267589

936

936

936

3211

3211

3211

412

412

412

137682915901

457151172

61083

61083

45114189

45114189

212235

212235

1

152025

5210

994246

1

1

914043

623426

53

24317

11

1

1

613

613

79661115647

51103

51103

36127

36127

22640

38

36

14613

6

27

3318424

2918361

25

438

23

23

189121

189120

1

1591102189

812146

71194

65266

3650

3115

13569

5833508

130

22

18

5622172

1

5664

11

365

104199

1311353472

1311353472

159637

159637

1414608

1414608

10

10

233103571

11657223

5623

11240325

1612007342

104561233

113

1277

39129

521325900

111

111

111

22

22

22

137341102239062

4335036144

7978804

16

3847492

4029301

115

27511228

1119232

22

7

1432987

2802813083

88217

6162688

6262517

1481451639

1

1421

4

4

47931025

47931025

428

428

428

4024752535

2983652279

3126

1211531453

174211800

104110256

8599184

191172

4250551

3338448

3338448

91199

3879

6320

14

14

235153130

217142113

1459673

15713

1

563927

181117

181117

2543671534

8528

8528

1086

1086

1

1

11

11

15106

15106

4

4

378027

13659

254

221014

23

23

1362381434

43

1292381429

32

524

524

13

13

310

310

36247

36247

2912368821146

2912368821146

1

3751148

2130136

151456

11095476

3213521455

2222041049

91014

2011276017106

4952253

117118453

1

751340801679

19541180805

19541180805

53922679837

14381060163

2095755501

1859864173

63

63

553219

553219

831589

2

831569

23289

23289

9126

9126

9126

8248663188

6051132

6051132

339330939

50109669

2273158

3854

111232

25312826

17954

17954

3434

13

12

1210

1

119

2352821346

437

1982331034

2129243

35

91757

3549171

3549171

8455

5353

11

211

1123256

1123256

688127

688127

361284

361284

81779

61475

234

349

349

12

12

482334558

232165294

232165294

250169264

250169264

10268229

8057212

7451208

664

221117

221117

5124211325

13895472

3524186

21

17

137

241526

2115

5238176

2

2

1

19751

241

151166599

5492366

19639

162073

1

6247121

121235

121235

361769

361769

12010764

12010764

3212

3212

501952

501952

2322

2322

1059

1059

1059

6523

6523

6523

6523

7782815141470

7782815141470

7782815141470

6416202825

10732

2573061116

3052326717647

138613806402

3524972393

157124907

3539328

316

11019914794

2292511658

2283261951

3313421411

3370

3370

3370

3370

64135604112299

27993915103868

35501449

5263

24401127

68221

38

3356

1329

1

226

97122

97122

1813452

1813452

541474237

24214

5

1322580

24

361153291

32129

214

81339

81339

85223

85220

1

2

1

1

1480237758509

210

26038710425

1

22441118

1572517361

3

2

1061634295

1

2151

1

3938518

2403918301

1115

4455

211

40774418077

1922455095

11

17

441053164

40763324578

7891

40762623687

5284208281

5

5284208276

1

1

75122

75122

2202154933

2202074773

8160

2227865

2227865

1611361789

26

26

5050565

2927219

141672

125

67249

8164680

342251

424

4340625

350

350

33

33

2420485

2420485

7029420

6632

6632

119

119

2

2

6219377

6219377

13

13

334814926057

1

1

2215

119

116

334614906041

289012665098

12

455224941

1

1

1

3532164

3532164

121845

2213102

1117

632245

632245

2

1

1

6828

115

3

1

12

2710

62

62

918

918

2

2

40133

1

1

1

40121

2

2

5959103

5959103

5959103

1

1

121

6616

5210

23

8

12

1

9425

22

1

182

1

122520

44

1

15512

8866853

8866853

8866853

1427

1

3315130

1

121068

1411157

2726470

5846201529

5846201529

5846201529

3924201105

374180

473686

108123258

4

4

4

4

4459132

4459132

3212

3212

91831

91831

2

2

133

133

1

1

152461

152461

1062

1062

2416

2416

115

115

1

1

11

1

1

341989

341989

3458

3458

261314

261314

5215

4113

112

2

2

27208

14135

14135

4

10125

1

1373

13

13

1211

1211

32

11

21

784758718129593875

19395120

1033847

1033847

1033847

885673

152211

152211

391815

391815

8821

8821

52

52

332

332

18324

18324

21

21

21

19088194

14459100

14459100

2

11

16

31

4927

21

2

1

6

1

753

11

314

752530

3

1

2

32

11

453

1134

33

2

2

1

11

214

513

5

1

1

1

201521

201521

201521

261473

231472

231472

31

31

401860438641361818

282532

6713

525

158

463

362

11

1279

1

1278

657

1

647

163811151124

22

22

32

32

288218195

715339

453918

606061

14

431

117

361814

24119

12615

781

936

1

7631

1196

1196

1563

1563

222128

161823

11

524

1

9168116

9168116

4

4

512

512

1306663

30628

2298

211

664522

131

1

922

1

1

42

42

352415

352415

1

1

21

21

43

32

11

12765214

12059213

761

2279

1778

2

21

1

9108

9108

279223111

11

1

1

13

276218110

1

1

1

2

2

744736

303412

421323

21

311

311

471538

471538

12110463

12110463

13610559

16116

1108750

821

252

936453

936453

1

1

261112

1996

726

1

1

853883

853883

4

4

396252435300356891

252714832236

431531

211512961944

19993114

22

1

16778143

12

393191432945354274

553377

432433326

21

111

155216131166

352518

121

111

15129

415846

11

391

13

111

1

14512870

390907430624352550

121

19491119

141118

1748099

62

212

212

338780260

338780260

23211

115

115

2

2

1114

1114

11

11

111

111

11

1

558330304

181416

181416

3567

3567

463274266

458271265

531

423615

423615

308217373349

326

326

521

521

1317

11

1

33

64

2

631

631

6051113

6051113

141012

141012

793391400

793391400

987

987

15596664

6757608

12

11

863854

241716

231

221415

1

1

9612061

9612061

2

2

41

41

71414

71414

718399716

718399716

685850

685850

371184324

7

355173317

2

1447

721

721

293156668

293156668

171110

171110

633278

633278

353173194

352173194

1

345

3

342

893832

23169

23169

561922

561922

811

811

22

22

1899374

1899374

381413

111717

22

1449

1

1235535

1

345671260403210244

14276149

14276149

14276149

140137359

136133358

136133358

441

441

231618

54

52

2

181218

181218

10857814756

7840121

7840121

551

25

31

211360

458

4634

13218

9817234574

428260631

10586268

3482652304

1001121371

771

551

351

2

22

22

869781206

1982

1982

711

711

51

51

2312

2312

272

1

262

691669139

691669139

554520

62

1

494418

831

831

784328

784328

211

211

1023088293254651

895537309738645

1

21

1

28715

222813

66446

11

18144163228269

232

2124

675215

461214

37208

472314

1

29412688

13496105

2097

15

434

532969

38

266528201055

2493

151

111

2

1922

1

371713

30141

1

162014

3

1

202713

11

211

191610

657835215028030

372713

311418

21136

172016

1157926

211

309155101

631

1245

3

3576122

1351803536

12

221710

1

17132

1

2

16112

1

1

1116363

1116363

340325728594

243038

310168442

1

679621727

1

32

593571

6239105

52

11

7453484532

41

3724151197

1

2117

235145262

2

314233475

442541

189162296

331332369

271014

11

876311104

876311104

640457706695

1137480

259214175

11

311262267

1

1

184200194

6959115

23

231811

515375

382435944117

16210

9087311230

211

1

1

494636

593512379

42214750

33

41914747

853310

853310

35116289

512838

30013451

1085764399

1085764399

51

51

51

10123762515219

348174213

348174213

2593921303

2593921303

32

32

9513705713703

9512705713702

11

18417112582662917

15278

8238

74

1

1

21

21

682849782416

682849782416

1

1

2

2

927220

11

24187

43

40306

31

1

62

72

11

12

11

875

1

6166

6166

623424

623424

17572211982059945

10101

1

17571211981059943

482121

301513

2

32

1

1347

917474

351

886973

1

1

1006608299

988598293

1896

1

11

1

1

181225

181225

26415272

914737

21232

1528233

1297

474

411

412

2305290116665

48

48

554

554

48427799

48427799

14

1

13

1379221215609

2262281057

410

914187214245

22

230110284

313

5654176

5654176

8411

8411

4710

4710

2

2

171148

171148

11

11

5515

5515

24112

24112

6563105

6563105

34

34

941

941

325

325

19623

19623

1

1

65

55

1

174200530

174200530

25

25

1

1

302613

302613

3

3

314245231

12724

12724

271221196

121

270219195

311711

311711

197111331371

778

778

196411261363

196411261363

202104121

115

115

1654

1654

1

1

1274

1274

342957

342957

12

12

1396238

1396238

1722081782

1

1

1632021763

1632021763

5417

22

3415

32

32

11

11

12897980510800

5356104

3

1

91220

414384

1579

2

1377

2

1

1

19712

19712

111713

111713

12249971741

12209961738

413

11

11

6155742001

6155742001

10174

10174

273274720

273274720

207153106

207153106

493

493

280269234

280269234

779451624207

11184

463637992951

314613451252

1

239122631644

238022381627

111

102416

363251261

431

431

3

3

656254

575752

852

632

632

534633

534633

223

223

1

1

511

511

643054

33

613051

161104103

161104103

823

823

823

713375352

633754

603443

3311

502669

502669

460228138

313917

1299991

3009030

13

13

1398191

1398191

5713

559

559

24

24

529402205

784228

1

784128

211

2

11

22616678

22616478

2

17615179

273424

14911755

474219

474219

921474224634

2098

3

1798

1002835384

1002835384

787439

787439

1

1

261918

1

51

201818

25148

25148

720528258

720528258

1

1

2

2

15118287

15118287

245155103

7127

23814396

986545

986545

9548113

9548113

1572

1572

550346643059

550346643059

1185387

1185387

764577335

577451229

187126106

31116

2

31114

7139

7139

862

862

19011341

18910941

14

1144930

1144930

486321117

1293

1293

2

2

2999

2999

716

716

24916673

151342

23415331

18913325

18913325

11

11

492349

431648

431648

641

641

3

3

93221033421527

93221033421527

196919712258

7353836319269

797159

797159

342944

106

21225

11

131310

792738

1

1

17

2

15

772731

581624

51

14116

6212

6212

23

212

217

303874

441

441

263371

52

3115

12

171861

1

11

11

12

12

12774114

513554

51313

27111

191140

4211

1

4111

1174

122

1052

96

94

2

625

625

462240

462240

335

222

222

1

1

11

11

2

2

1

1

1

181815

181815

181815

11

11

11

286194155

286194155

286194155

472443

11

11

462342

462342

1

1

7572823613321

321227376

321227376

7251800912945

5562644710279

102810011582

6615611084

8476961500

444353900

159134442

159134442

11680141

11

11919

4

1

9611

6116

13

839

1

8412

12

783

594964

9284172

9284172

6451124

5746106

7518

13421

16

12415

7256191

7256191

7256191

331287409

4218

4218

327285391

327285391

1

1

1

1

220981110111644

20245982710580

374168263

374168263

1276166948155

1254

9944103

1264666448045

1

313

711029652162

711029652162

70288

70288

1316

57272

1026653778

22513753

22513753

801515725

16851170

633464555

1

1

757593278

501494229

501494229

422912

422912

2137037

2137037

1

1

11

11

11

11

1227462696203

237114152

237114152

11

12

1048572

826

11

1

1

11

23

1

966

1

10210

122

8

731

28521

42

50625

930280643

927280643

912275602

15541

3

3

14165129

14165129

1

9647102

25819

1794

214

32572228

32572228

414

527

31669217

1042351294927

1247

2

222

1

611

1

12

11

15290129

12169

2

3

464156

792147

323

1

7911

3

1354

121

31

831

11

562172

562172

1610750378

1571702366

374812

2

468520462027

1936187

1365338

392517641741

233

27207

9

402136151

402517

402517

4659469

24814

2156445

2232010

32

491738

491738

17219941132

54

2655

11

15548991045

814659

1

1

1

543821

1188851

1188851

281561

281561

721

721

1462975940

1461974940

1

1

1

1

431

331

1

217609124

217609124

217609124

1

1

1

776466

776466

776466

776466

42

42

42

42

194108768

194108768

11387724

11387724

812144

812144

12786281270

322

322

1

321

33290223

33290223

33290223

11978240

11978240

11978240

714367362

16418

16418

741

741

1034

1034

1076

1076

2

2

51420

51420

225113121

225113121

2124

2124

52

52

16144

16144

1

1

723451

723451

561816

561816

35237

35237

31

31

1171

1171

1079

1079

1579287

1579287

23

23

50248

50248

2936307

2020117

1099

3

1011105

916190

916190

8155136

1

1

8154136

8154136

181517

181517

181517

181517

1

1

1

1

531730

531730

441221

1

421118

1

211

959

959

1045453

1045453

804749

804749

924

332121

1356

251918

2474

2474

2474

10417924

10417924

10417924

10417924

10417924

21131541605

21131541605

22411166

985837

2

1

2

121

1779

1

1

812

1

3

12

236

1

1054

392

2392

8717

1

1

1321

1321

934725

1

1628

774417

2043

1213

1

1

53

1

179194113

145184100

13717995

521

334

341013

341013

394732

14

1

1

12

2131

2121

1

1666

51

1156

8182

1

2

86

1

21

1

6

13919

1

29

1019

16612

16612

16612

30611

30611

1934

62

1

534

461

461

21

44

252232119

131

1

1

12

582128

371419

1

2169

635917

1

261810

26146

10271

28621

832

3

1

1

18314

1

1

10171

1

1

32

4

1

1

1

181

4

141

141

221312

211012

11

2

311

1

39

1

26

26

191210

16124

36

458028

297422

32

5

1314

46035954

723

1

21

31

4

2244

2244

26425810

26425810

1679537

41

731

50335

522

2

1

11

1

28217

34

592419

321

1

231

11

3

3

1

2

19920632

1

1

17617928

1

2

2

1

515310

3

11812316

2

1

11163

3

8163

12101

23

1

1

2

61

14

11

685366150

321

1

111

11

3

2

1

1

1

46811

4487

1

14

54129959

231

53829558

11

361

361

1

1

7735

7735

3232

3232

12

1

2

762139

11

3826

1111

1

2

121411

321

514

113

41

22815

2071

2

2

11

1361

2

2114

1

214

876332179

415

415

41

11

3

5

1

4

1

1

1

1

62

62

62

62

30812

30812

2438

1527

911

654

243

411

1

1

1

1

1667653

11

11

11

1586550

1382

1382

422

422

34178

34178

1073838

1073838

8102

42

42

41

41

45

45

1153

332

332

332

821

321

321

5

5

62226

62226

62226

62226

107

107

107

107

28308

28308

20212

20212

896

896

1

1

1

1

11111

11111

11111

11111

40913650

77

77

1

67

562311

5

4

1

512211

1

114

871

1

92

32106

1

1

1

1

1

1

611

611

611

1

1

1

12

12

12

52

1

1

42

42

33410037

681212

4598

2334

143

91

41

11

2528525

2528525

581412

581412

11

11

11

11

124

1

123

1022

1022

1

1

23

2

21

2

2

21

21

336

336

21

1

1

1

321

321

131411

131411

13

13

121111

121111

66167

1741

1741

1741

49126

5

5

44126

44126

9136103565727

9136103565727

9136103565727

783612

103

3

653312

1

1

632374662569

632374662569

651

651

114

114

492184

1

482184

494015

494015

19052438823

19052438823

2531417

5116

2030401

281513

281513

221114

221114

469100163

469100163

211442

211442

1

1

11

11

3

3

2619

2619

341110

341110

55601515

55601515

353411

353411

32644

32644

3849241356135790

1095

695

12

12

683

441

242

4

4

4

182741

182741

182741

161540

2121

773205140

773205140

2669

1

2659

56413286

44

52812375

2294

103

1836745

1836745

471619

471619

1

1

124

124

713

713

1

1

23107

23107

1132

1111

21

11

11

4

4

3330938615129310

2999935745120648

199732669781169

8149

1928194

12

1516310

1218

99134328

2813

2

4054963102

1

4731473526625

203138

313

24

11

12

1

2519

343

5310

51418

7119

1

4936308

1110

2610

161816

65472

12717

1

1

231128

24

112

7073918

212

3318

6

11

3413

2111105

311

4476161

200674224

113

13

12

1

111224

1911781988

8711

4168370

11

92874

1

2417

12

221519

59110391511

5512

35

2

112

3217

32

210

11680385

652

1

312

117

6715

212

313

139121143

3754254

12

114

5742101

24424

11

3532336

7723

1

3818

21

1518191

17

1

2417

25511

16

101160

2

2333292

2

23

3085991788

452

534848

662303633

211

12213

16

21

101054

5429

7166

7139

144449

8

1

5443112

1

6113

171545

27

11

1

175183536

107153534

192228

106861600533449

344341

131612

1

36

2

2210

61647

101262

11

9816

12

3836974229

1

3437

1911

2

1

1

44

77

3126118

127

3104

272972

11

4120

201233

1

1

21

1

161585

11111

17282

24

1213

121

111445

4419

6428

112

219

13

1242

1242

243196699

243196699

369437

369437

9737874038666

1

6716

5817

411

231519

1212120

173139226

1310

5655151633

9214

7461242

19317

1

11

1146

2232146

18

189166530

1

1215

411

355118

11

15

242426

5243117

3112

1

238323573285

1

1

2619

9873741

4213

292118

292388

81253

133119392

3513

330320582

191134525

555

511

108108

15834

181416

3

1

5917

21

6524232929

5714

675681

1254

332649

12

114

2027182

1

121429

3

2524

191834

111238

4312

16824

191746

78105104

11

23

464

1

2926

191681

445

3765122577

27646712207

11613

61680

122102497

222

5510

3221881733

5

295238497

3411

412

13

5150130

214

5715

8646941880

3349

303289

12

24816

1

6303772168

5423

9797246

2

11795111

271063

618

1

101137

537

5157101

231

22

114

151827

1

274

4743181

9139

182062

141335

938

878

212

1

91135

8714

31

1

9818

167122666

16599976

13822

304063

7415

71114

246

401988

224912

10250417

11

265

134

9137

101049

265052

15

11

5511

3668205

2

24

2

71727

11

1

3641

1

111

8213

112

6414

6122

3

1313

211963

91334

12

1

339

6922

31

31

319527047595

3013361602

3013361602

4058275

227

134

1730123

1221

121472

14

7734

285423105716

7866105

1

212151515

1

21933

6477

91122

12

2612

151516

1

856526475

1

121237

311

3747113

1

716

336

12068538

324

111

101326

3830133

10489172532

1

4421

222614

644

418

884

1

233143

2327106

6279

211213619

232

2110

21

263147

314072

122

1121

13

1411

3

363

2

2

8199361

131041

34

10933

14

4357180

4357180

2532140

222594

33

25

2

1336

1

3467706

3467706

3467706

7388863974

2783621624

34

34

71521

71521

333

333

113166558

2

4

1917121

2

6108

2362128

11528

2323120

218

172789

3210

91740

5633

1

4633

326

326

138158879

138158879

1151

1151

1

1

3213

3213

5826

5826

121172226

5543

1121

4422

6

6

211

211

17513

12

16511

97161163

97161163

1

1

1

1622301129

1602291108

123

11

5476624

1965

95132370

232

1

6524

218

2121

2121

3432254

1711141

2

11

23

1151

1

3132

6

2518

2210

418

210

3

3

311

211

1

91341

91341

1

1

510

4

1

55

3547

1

3

1

341

21

111

11074597

5323

5323

10571574

10571574

281376

23768

23766

2

568

12

1

1

11

1

22

313

5367

5367

1

6

352

2

2

34

2

643

541

541

541

12

12

12

719167

719167

719167

719167

355415572067

1335630554

2216840

2216840

431207245

431207245

936179

523

885976

590294190

652525

1525

506266160

41

703659

261113

2145

2

576

6

6

382546

382546

1254736

1224536

42

821

1

451511

371413

1

1585

1

1154

32

32

17397081177

435

435

15753126

15753126

1407257

912623

2

474634

24573130

14738103

892922

965

1193507859

976320558

41

140163259

491333

742

32

1744

285136241

131

1

21

1

492640

31

9917

371623

213

213

41

41

13424

11424

1

1

1

1

23

22

1

763349

1

49

14914

11

19814

11

1322

25137

441

441

13362118

161426

68

20

592923

2281

642

33

7115

1

15

312

222

2

72

1

22

22

301864

301864

81018

81018

12638

13

11635

317

317

711

711

11267218096

11267218096

121835

2728

1

1

1

1

325

13

1

10117

10117

2

2

2

584309622

495267417

434203380

606237

12

71

6

11

8242204

22

51

7541202

11

11

11

4252786737

26838

26838

998

25

34

6

42

753974

753974

3152226617

3142186599

1418

8899651

1828

1828

3130231

3130231

2925144

2925144

2736248

2736248

141651

141651

1139

131512

804252

804252

27932

1829

1829

9723

9723

533320

647

646

1

35

35

32264

821

24243

31

31

933

2

733

23054563

22752561

929

22

22

22

79

79

79

19643535

19643535

19643535

1

18440516

12318

20715

42

31

31

11

11

3

3

3

11

11

11

712

712

712

536

536

536

331

331

331

22

22

2

2

2

2

322

322

2

2

2

32

32

32

1329122025284

5514111065

17945342

17945342

17845342

1

1

11

1

32

115

21

1129

3

1

124

78

857

312

1

83128

18737

3

26

18

326

113

861863

1111

25

5410

1

1

624

624

624

4

410

1

110

422

222

222

222

2

2

2

362364697

674

644

111

4

33

1

3

3

11

11

11

112278

81571

81571

376

1

3

312

51

1

1

265307375

265307375

2

1

256294343

91329

5

5

5

14

14

4

1

1

1

1

1

1

1

7823233

116

116

7722227

2

1

4812133

928

9453

1

11510

77880924219

77080924209

37178121

573

573

36671118

36671118

17

17

17

39873124081

39873124081

39873124081

81

5

3

3

2

2

31

31

31

9

9

9

9

113

113

113

113

113

1

112

6327

6327

6327

313

313

313

3224

3224

3224

11118

11118

11118

11118

11118

91

2117

391437

381130

381130

381130

381130

36817

2313

137

137

137

137

1

136

11846

11846

11846

11846

11846

11846
